# Characterization of novel CTNNB1 mutation in Craniopharyngioma by whole-genome sequencing

**DOI:** 10.1186/s12943-021-01468-7

**Published:** 2021-12-18

**Authors:** Juan He, Zhen Zeng, Yuelong Wang, Jiaojiao Deng, Xin Tang, Fujun Liu, Jianhan Huang, Hongxu Chen, Ruichao Liang, Xin Zan, Zhiyong Liu, Aiping Tong, Gang Guo, Jianguo Xu, Xiaofeng Zhu, Liangxue Zhou, Yong Peng

**Affiliations:** 1 Laboratory of Molecular Oncology, Frontiers Science Center for Disease-related Molecular Network, State Key Laboratory of Biotherapy, West China Hospital, Sichuan University, Chengdu, 610041 China; 2 Key Laboratory of Bio-Resource and Eco-Environment of Ministry of Education, College of Life Sciences, Sichuan University, Chengdu, 610064 China; 3 Department of Neurosurgery, West China Hospital, Sichuan University, Chengdu, 610041 China; 4 Department of Neurosurgery, Huashan Hospital, Fudan University, Shanghai, 20040 China

**Keywords:** Craniopharyngioma, WGS, β-Catenin

## Abstract

**Background:**

Craniopharyngioma (CP) is rare histologically benign but clinically challenging tumor because of its intimate relationship with the critical structure in the central brain. CP can be divided into two major histologic subtypes: adamantinomatous-type CP (ACP) and papillary-type CP (PCP). Although some genetic aberrations for both categories have been revealed in previous studies, the complete spectrum of genetic changes of this tumor remains unknown.

**Methods:**

In this study, we conducted whole genome sequencing (WGS) on twenty-six CPs including 16 ACPs and 10 PCPs together with their matched blood samples. Somatic variants (SNVs, InDels, SVs and CNVs) were identified and mutational signatures were characterized for each patient. We investigated the impact of a novel CTNNB1 mutant on its protein stability, ubiquitination and Wnt pathway activity. Cell proliferation ability of the CTNNB1 mutant in ACP primary cells was additionally analyzed by CCK8 and colony formation assays.

**Results:**

We found that CPs had showed less complexity with fewer somatic mutations compared with malignant tumors. Moreover, mutations in CTNNB1 (68.75% of ACP) and BRAF V600E (70.00% of PCP) are mutually exclusive in ACP and PCP, consolidating that the driving roles of these two genes in ACP and PCP, respectively. A novel mutation in the exon 3 of CTNNB1 which compromised both a transversion and in-frame deletion was identified in ACP. This mutation was experimentally validated to confer β-catenin increased stability by inhibiting its ubiquitination, thus activating Wnt-signaling pathway and promoting cell proliferation.

**Conclusions:**

Whole genome landscape for CP was revealed by WGS analysis, and a novel mutation in the exon 3 of CTNNB1 was identified. This novel mutation activates Wnt-signaling pathway through increasing the stability of β-catenin. Our findings provided us with more comprehensive insight into the spectrum of genetic alterations in CP.

**Supplementary Information:**

The online version contains supplementary material available at 10.1186/s12943-021-01468-7.

## Background

Craniopharyngioma (CP) is a rare primary type of benign brain tumor with incidence rate of 0.16 per 100,000 [1, 2]. CP develops in the sellar region adjacent to many important functional structures, such as optic nerve, optic chiasm, pituitary stalk, hypothalamus and internal carotid artery [3]. Surgical resection is the primary treatment for CP currently. Gross-total resection (GTR) for CP has been the preferred treatment but with high rates of optic and endocrinological impairments [4]. While patients who undergo sub-total resection (STR) are often consequently at the risk for recurrence. Overall, CP is characterized by “the most formidable of intracranial tumors” with a high disability rate, leading to poor quality of life and an increase of mortality rate on long-term follow-up [1, 5].

The two histological subtypes of CP, adamantinomatous-type (ACP) and papillary-type (PCP), differed in their age distribution, pathological characteristic and genesis [1]. ACP is the more common subtype affecting all age groups, whereas PCP is mostly restricted to adults. ACPs are predominately cystic and show typically prominent calcifications [6], while the typical PCPs are more frequently non-calcified and ‘solid’ [7]. Through target sequencing or whole-exome sequencing, recent studies show that the two subtypes have distinct molecular pathogenesis mechanisms [8–10]. ACP is driven by somatic mutations in CTNNB1 gene (encoding β-catenin), and most of these mutations are point mutations within the exon 3, thus affecting regulatory amino acid residues involved in β-­catenin protein stability [11, 12]. Mutated β­-catenin cannot be efficiently degraded, leading to its nuclear accumulation and the activation of Wnt signaling pathway, which is critical for tumor development. For PCP, no other recurrent mutations or genomic aberrations have been identified except for the somatic BRAF V600E [13]. This mutation was observed in the majority of PCPs to activate MAP kinase/ERK signaling for tumor progression.

Because target sequencing or whole-exome sequencing may miss key insight into some important genome region, the complete spectrum of genetic changes of CPs remains undefined. In this study, to better understand genomic alternations of CPs, we conducted whole-genome sequencing (WGS) of 26 CPs including 16 ACPs and 10 PCPs, along with their matched normal blood samples. We found that there were fewer somatic mutations in the CP genome compared with other malignant counterparts. Moreover, we confirmed that mutations in CTNNB1 and BRAF V600E were mutually exclusive in ACP and PCP respectively. Interestingly, we identified a novel mutation of CTNNB1 gene in ACP and experimentally characterized its oncogenic functions in vitro. Therefore, our study provides more comprehensive understanding of the genetic spectrum in CPs.

## Materials and methods

### Clinical samples

This study was approved by the Institutional Review Board (IRB) of West China Hospital (File No. SKLB20140830–02), China, and informed consents were obtained from all patients. Samples were collected following the relevant ethical regulations for human participants. Patients histologically diagnosed as craniopharyngioma in West China Hospital (China) from January 2014 to July 2015 were included in this study. Samples were immediately snap-frozen on liquid nitrogen and stored at − 80 °C until needed. This study cohort comprised a total of 26 patients (Additional file 1:Table S1) including 16 ACPs and 10 PCPs. Age and gender distributions of these samples were shown in Additional file 2:Fig. S1. Genomic DNAs were extracted from tissues and blood samples using QIAamp DNA Mini kits (Qiagen). DNA concentrations were measured with NanoDrop 2000 (Thermo Fisher Scientific).

### Whole genome sequencing

Whole genome library construction (5 μg DNA) and sequencing were carried out by WuXi AppTec, China. Briefly, DNAs were sheared with Covaris S220 Sonicator (Covaris) to a target size of 300–400 bp. Fragmented DNAs were purified using Sample Purification Beads (Illumina). Adapter-ligated libraries were prepared with the TruSeq Nano DNA Sample Prep Kits (Illumina) according to the manufacture’s protocol. DNA concentrations of the sequencing libraries were measured with the Qubit 2.0 fluorometer dsDNA HS Assay (Thermo Fisher Scientific). Quantities and sizes of the sequencing libraries were analyzed using Agilent BioAnalyzer 2100 (Agilent). The libraries were used in cluster formation on an Illumina cBOT cluster generation system with HiSeq X HD PE Cluster Kits (Illumina). Paired-end sequencing was performed using an Illumina HiSeq X™ Ten for 2 × 150 paired-end sequencing (~ 30 X coverage, ~ 90 Gb raw data per sample).

### Data processing and detection of somatic variant

Raw reads were converted to FASTQ and then aligned to human reference genome (hg19) with BWA-MEM algorithm [14]. Duplicated reads removal, local realignment and base quality adjustment were performed with Genome Analysis Toolkit (GATK 3.8) [15]. Depth and coverage of sequencing data for each sample was described in Additional file 1:Table S1.

To establish highly sensitive somatic variants sets, base substitutions were initially called by Mutect2 [16] and Strelka2 [17], while InDels were initially called with Strelka2 and Varscan2 [18]. Then the resulting initial somatic variant sets were firstly filtered using locus background constructed by WGS data of normal blood samples. Final somatic variant sets should meet the following criterions: 1) mutation rate of the locus should be not less than 10%; 2) base quality ≥20. In addition, for somatic SNVs, the number of the reads span the locus should be over 14/8 in normal/tumor samples. The reads depth of InDel locus should be over 10, and the *p-*value of InDels called by Varscan2 should be ≤ 0.001. Mutations in low complexity regions such as tandem repeat regions were filtered out. Finally, the passed somatic variants called by Mutect2 were all retained, while the variants called only by Strelka2 or Varscan2 were filtered. Manta was used for somatic rearrangement calling, supplemented by structural variation calling method by Lee et al. [19], then Integrative Genomics Viewer (IGV) was used to visually check the rearrangements, removing the false positive events. Sequenza was used to estimate tumor purity and ploidy [20].

### Kataegis analysis

Kataegis analyses of total small mutations (SNVs and InDels) were performed with the R package of regioneR and karyoploteR, different types of mutation were plotted with given color; C > A was in purple, C > G was in red, C > T was in sky blue, T > A was in forest green, T > C was in orange, T > G was in navajowhite and others including insertion and deletion were in grey.

### Mutational signature analysis

Mutational signatures were analyzed with DeconstructSigs [21]. COSMIC signature 1/5/6/11/14 and signature 1/5/8 were previously identified and validated in adult and pediatric brain cancer respectively. The relative contributions of these six mutational signatures were firstly calculated for each sample. As there was no analysis on CP WGS before, all the 30 COSMIC signatures were taken into account to compute their contributions to CP.

### Primary cell culture

Fresh ACP samples were washed with PBS (pH 7.4), minced into pieces of 2–3 mm in size, and enzymatically digested with 1 mg/ml collagenase (Sigma-Aldrich) for 40 min at 37 °C in an incubated shaker under sterile conditions. The collagenase was inactivated by adding DMEM-HG with 10% fetal bovine serum (Gibco) and 1% penicillin/streptomycin. After centrifugation, cell pellets were resuspended in EpiLifeTM medium (Gibco, cat# MEPI500CA) with human keratinocyte growth supplement (HKGS) (Gibco, cat# S0015), followed by incubation in 37 °C incubator with 5% CO_2_.

### Plasmid construction

To construct β-catenin expressing plasmid, cDNAs were reversely transcribed from total RNAs of patents’ tissues and then subjected to amplifying the full-length of open reading frame (ORF) encoding β-catenin by Phanta Super-fidelity DNA polymerase (Vazyme, China, cat# P501-d1) using the forward primer (5-GATTCTAGAGCTAGCGAATTCGCCACCATGGCTACTCAAGCTGATTTGATG-3) and the reverse primer (5-GATCCTTGCGGCCGCGGATCCTTACAGGTCAGTATCAAACCAGGC-3). Employing ClonExpress™ II One Step Cloning Kit (Vazyme, China, cat# C112–01), the resulting ORF fragments (wild-type or mutant) were recombined with the linear vector pCDH-CMV-MCS-EF1-copGFP digested with *EcoR*I and *BamH*I to obtain the recombinant plasmids designated as pCDH-CTNNB1-WT or Mut. For knockdown of human β-catenin expression, two synthesized oligos (5-CCGGGCTTGGAATGAGACTGCTGATCTCGAGATCAGCAGTCTCATTCCAAGCTTTTT-3 and 5-AATTAAAAAGCTTGGAATGAGACTGCTGATCTCGAGATCAGCAGTCTCATTCCAAGC-3) were annealed and inserted into the pLKO.1-TRC vector at *Age*I and *Eco*RI sites. To express wild-type or mutant β-catenin in CTNNB1-shRNA knockdown cells, the β-catenin-expressing plasmids prepared as above were subjected to synonymous mutations by QuikChange II Site-Directed Mutagenesis Kit (Agilent Technologies, cat #200524) and these primer sets (5-GAGCCAATGGCTTGGAACGAAACGGCTGATCTTGGACTTGAT-3 and 5-ATCAAGTCCAAGATCAGCCGTTTCGTTCCAAGCCATTGGCTC-3).

### Cell culture and virus preparation

HEK293T and HCT116 cells were cultured in high glucose DMEM media (Gibco) supplemented with 10% fetal bovine serum (Gibco) and maintained in 37 °C incubator with 5% CO_2_. To generate lentivirus expressing β-catenin, HEK293T cells grown on a 6 cm dish were transfected with 4 μg pCDH-CTNNB1-WT or Mut, plus 3 μg psPax2 and 1 μg pMD2G. 72 h after transfection, the supernatants containing the lentivirus were collected. To establish stable cell lines, cells were infected by different lentiviruses and selected by puromycin or GFP cell-sorting depending on the lentivectors used.

### RT-qPCR

Total RNAs were isolated by RNAiso Plus reagent (TaKaRa, cat# 9108) according to the manufacturer’s instructions. To measure mRNA levels, cDNAs were generated from total RNAs by PrimeScript RT reagent Kit with gDNA Eraser (TaKaRa, cat# RR047A), and quantitative real-time PCRs were performed with TB Green® Premix Ex Taq II (Tli RNaseH Plus) (TaKaRa, cat# RR820A). ACTB mRNA was used as endogenous control, and the fold changes were calculated using the 2^-ΔΔ*C*t^ method. The primers used were as follows: CTNNB1 forward: 5-GAACTGTCTTTGGACTCTCAGG-3, reverse: 5-TGCACAGGTGACCACATTTA-3; SOX9 forward: 5-CTGGGCAAGCTCTGGAGAC-3, reverse: 5-TTCTTGTGCTGCACGCGC-3; c-Myc forward: 5-CGTCTCCACACATCAGCACAA-3, reverse: 5-CACTGTCCAACTTGACCCTCTTG-3; and ACTB forward: 5-AGGCCAACCGCGAGAAGATG-3, reverse: 5-GCCAGAGGCGTACAGGGATA-3.

### Western blotting

Proteins were extracted with RIPA lysis buffer containing protease inhibitors and phosphatase inhibitors, and their concentrations were measured by BCA Protein Assay Kit (Beyotime, China, cat# P0010). Following separation by SDS-polyacrylamide gel electrophoresis, proteins were transferred onto PVDF membrane (Millipore) using Bio-Rad Mini Trans-Blot System. Then membranes were blocked for 1 h with 5% non-fat milk in TBS-T buffer at room temperature and incubated with primary antibody solutions at 4 °C overnight. Blots were washed with TBS-T (three times, 5 min/wash) and subsequently incubated with horseradish peroxidase (HRP)-conjugated secondary antibodies at room temperature for 1 h. After washed with TBS-T three times, blots were incubated in SuperSignal™ West Dura Extended Duration Substrate (Thermo, cat# 34075) and imaged by Bio-Rad imaging System. Primary antibodies used in this study were as follows: rabbit anti-β-catenin (Cell Signaling, cat# 8480), rabbit anti-phospho-β-catenin (Ser45) (Cell Signaling, cat# 9564), rabbit anti-SOX9 (Abcam, cat# 3697), rabbit anti-c-Myc (Abcam, cat# 32072), mouse anti-ubiquitin (Cell Signaling, cat# 3936), and HRP-anti-Actin (Cell Signaling, cat# 4970).

### Immunoprecipitation (IP) analysis

After washed twice with cold PBS, cultured cells were collected and resuspended in RIP buffer (200 mM Tris-HCl, pH 7.5; 20 mM MgCl_2_; 300 mM NaCl; 10% glycerol; 0.5% NP-40; 0.5% Triton-X 100) plus protease inhibitors and phosphatase inhibitors. Then cell suspensions were incubated on ice for 10 min and lysed by sonicator. After centrifugation at 14,000 × g for 10 min at 4 °C, cell lysates were incubated with primary antibodies or control IgG overnight at 4 °C, and further with protein A beads (Millipore, pre-treated with 5% bovine serum albumin in RIP buffer) for another 4 h. The beads were washed three times and boiled for 5 min in SDS-PAGE sample buffers to elute bound proteins for Western blotting.

### TCF/LEF reporter assay

Cells were cultured in a 96-well plate until reaching ~ 80% confluency, and then transfected with TOP-FLASH and FOP-FLASH reporter plasmids together with the Renilla luciferase control vector pRL-TK. After 36 h of transfection, luciferase activities were measured with Dual Luciferase® Reporter Assay System (Promega, cat# E1910). The Firefly luciferase signal was normalized to Renilla luciferase signal, and luciferase activity data were standardized to each condition by calculating the TOP-FLASH/FOP-FLASH activity ratios.

### CCK-8 assay

Cell viability was measured using Cell Counting Kit-8 (Beyotime, cat# C0037). Briefly, 3 × 10^3^ cells were seeded into each well of the 96-well plate and cultured at 37 °C overnight. At the indicated time points (1,3,5 and 7 days), 10 μL of CCK8 was added to each well. After 3 h incubation, the optical densities at 450 nm were measured by a microplate reader.

### Colony formation assay

ACP stable cells (9 × 10^3^ cells) were plated into each well of a 12-well plate and cultured at 37 °C for 10 days, with adding fresh growth medium every 3 days. Cell colonies were fixed with 4% paraformaldehyde and stained with 0.5% crystal violet solution.

### Immunohistochemistry

The tissue sections were incubated in an oven at 65 °C until the paraffin melts, followed by deparaffinization in 100% xylene and dehydrated with graded alcohol solutions. Antigens were retrieved in a 10 mM sodium citrate buffer (pH = 6.0, ZSGB-BIO, China) preheated to 95 °C for 10 min. The tissue samples were naturally cooled to room temperature (RT) and incubated with 0.3% H_2_O_2_ for 10 min to reduce endogenous peroxidase activity. After washing three times in phosphate buffered saline (PBS, pH 7.2), these sections were blocked by 5% goat serum for 15 min. For β-catenin detection, blocked sections were incubated with anti-β-catenin antibody (1:50, Cell Signaling, cat# 8480) overnight at 4 °C in a wet box, then washed three times in PBS and incubated with HRP-conjugated anti-rabbit antibody (1:1000, ZSGB-BIO, China) at 37 °C for 15 min. The sections were stained with DAB^+^ substrate-chromogen solution (Maixin Biotech, China) at RT for 30 s. After rinsing with distilled water, the sections were counterstained with hematoxylin and subsequently dehydrated, mounted and covered with coverslips. Normal blocking serum without primary antibody was used for the negative control. Images were acquired with a Pannoramic MIDI Slide scanner (3D HISTECH, Hungary).

### Fluorescence in situ hybridization (FISH)

The tissue sections were preheated in a 60 °C oven for 50 min until the paraffin melts, and fully dewaxing in fresh TOI and TOII (clearing agents) for 10 min each. Next, tissue slices were re-hydrated in RNase-free gradient alcohol (100, 95, 90, 80, 70%) for 10 min each. Subsequently, wash the slices twice with PBS for 2 min and performed the following experiments using Ribo™ Fluorescent in Situ Hybridization Kit (RiboBio, China) according to the manufactures’ instructions. Briefly, the sections were exposed to 0.5% Triton X-100 for 5 min, prehybridized at 37 °C for 30 min, and then in situ hybridized at 37 °C overnight with Cy3-labeled probes detecting FBXW7 mRNAs (5-TACAAGCCCAGTGGTACTTGTATATTCTGAG-3, and 5-ATGTTCTCAGACATTTGCCTGTGACTGCTG-3). After extensive washing, nuclei were stained with DAPI. The imaging was performed on a Nikon structured light-Illuminated confocal microscope.

## Results

### Summary of somatic mutations of CP genomes

**Fig. 1:**
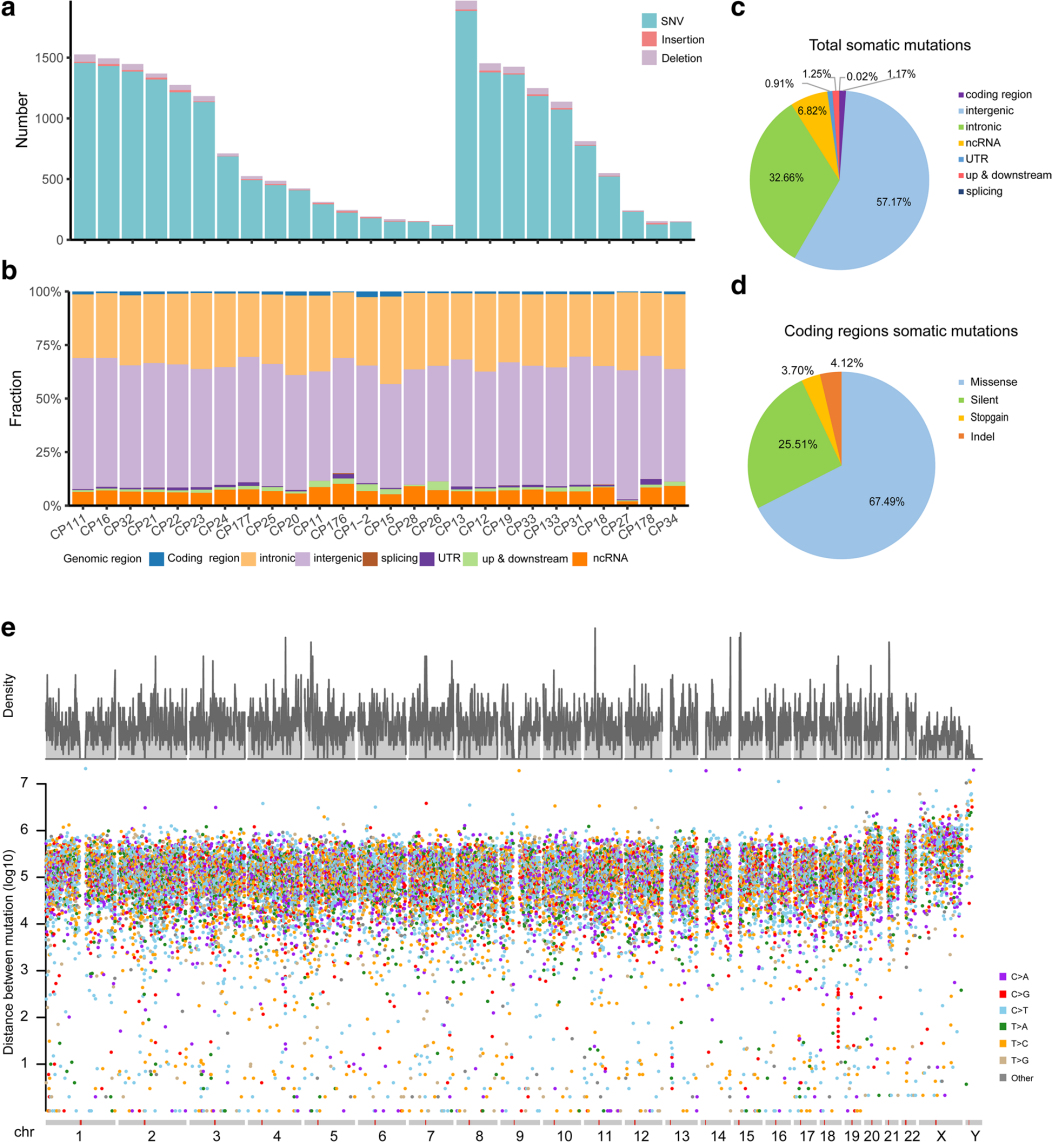
Summary of somatic alterations in craniopharyngioma samples. (**a**) The total number of somatic SNVs/InDels identified in each CP, (**b**) proportion of somatic SNVs/InDels located in different genomic regions for each CP, (**c**) proportion of different regions of all somatic SNVs/InDels, (**d**) proportion of different types in coding regions. (**e**) The rainfall plots of all somatic mutations (SNVs and InDels) of 26 CPs, the x axis shows the chromosomes of human genome, the y axis represents the density of mutation in one position (up) and the genomic distance (in log scale) of each mutation from the previous mutation (bottom)

In total, 26 CPs including 16 ACPs and 10 PCPs underwent whole genome sequencing, with a median read depth of 29.51× (range 22.75–34.91) for tumors and 29.75× (range 22.97–34.52) for blood samples after removal of PCR duplicates (Additional file 1:Table S1). We detected 20,772 somatic mutations including SNVs (single nucleotide variants) and InDels (Insertions and Deletions) with an average of 760 SNVs and 39 InDels per patient (Fig. 1a and Additional file 3:Table S2). We also found that total somatic mutations significantly increase with age (Additional file 2: Fig. S2, *p* value = 0.02528, r = 0.44 by Pearson’s correlation test), consistent with previous reports [22, 23]. Jackson et al. [24] proposed that DNA repair machinery may be attenuated with age. The average tumor mutational burden (TMB) for SNVs and InDels were 0.246 bp/M and 0.013 bp/M, respectively. Compared with large cohorts of other tumor types [25], the relatively lower TMB in CPs may be consistent with its benign histology. Most of these mutations occurred in the intergenic regions, followed by the intronic regions (Fig. 1b and c). While only 243 somatic mutations (1.17% of total somatic mutations) occurred within coding regions linking to 211 genes. Among them, 164 (67.49% of 243 exon somatic mutations) were missense mutations, 62 (25.51%) were silent mutations, 9 (3.70%) were nonsense mutations and 10 (4.12%) were InDels (Fig. 1d). The average number of protein-coding mutations in CP was 9.35 (range 1–26). A relatively smaller number of SVs (Structural Variants, 5.69 per patient) were identified in CPs (Additional file 4:Table S3; Additional file 2:Fig. S3), which may also be due to CP’s benign histology. Interestingly, kataegis analyses of all somatic SNVs and InDels showed that the mutations tend to occur at the terminals of chromosomes (Fig. 1e and Additional file 2:Fig. S4). This phenomenon has not been observed in other malignant tumors, which may be a consequence of large number of somatic mutations across whole chromosomes in the genome of malignant tumors. Moreover, these frequent somatic mutations identified in the chromosome ends of CP genomes were close to the telomere regions (the telomere sequences are the gap in chromosome ends of the reference genome). Given that telomere region is frequently affected by DNA damage during aging [26], our results suggest that the regions close to telomere also tend to suffer DNA damage in the benign tumor. Interestingly, one kataegis loci not close to chromosomal terminal was also found on chromosome 18 (q22.1: 66258699–66,261,950, intergenic) of CP13 (Additional file 2:Fig. S4).

### Mutational spectra and signatures

**Fig. 2:**
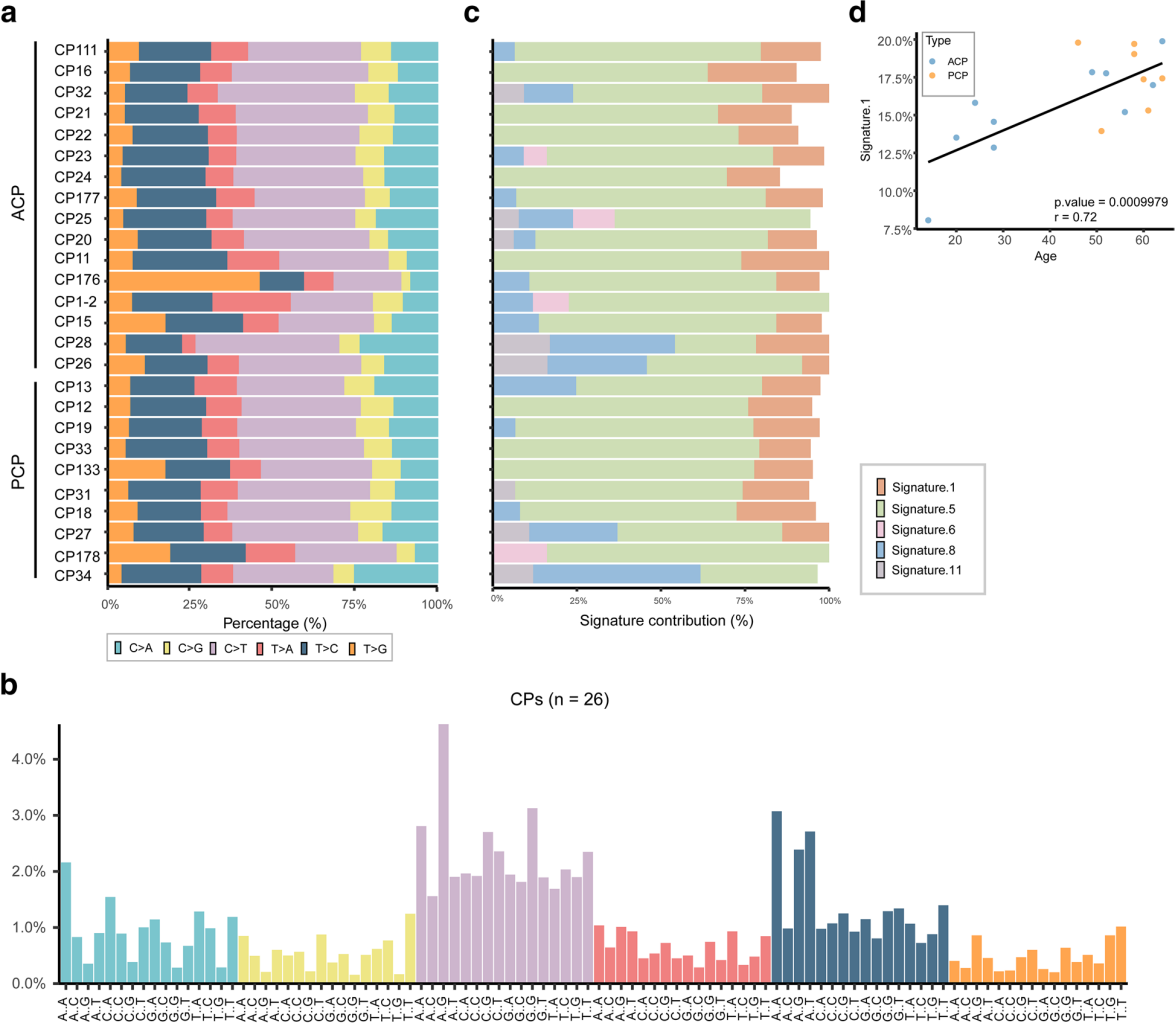
Representative mutational spectra and signature contributions to CPs. (**a**) The relative proportion of six different possible base-pair substitutions in each sample, (**b**) the proportion of 96 different trinucleotide substitutions of all SNVs identified in 26 CPs, (**c**) five signatures’ contribution for each patient in our study cohort. (**d**) Correlation between content of signature 1 and age at diagnosis of each patient (17 CPs with 0 < signature 1 content < 20%), Pearson’s correlation test was performed

Single base substitution is classified into six categories: C:G > A:T, C:G > G:C, C:G > T:A, T:A > A:T, T:A > C:G and T:A > G:C. It was found that C:G > T:A transition, a signature of mismatch repair deficiency, occur dominantly in many cancer genomes [27–30]. We performed genome-wide mutational spectra of CPs and found that C:G > T:A transitions (36.57%) and T:A > C:G (22.03%) transitions were the dominated substitutions in our study cohort (Fig. 2a and b).

The 5′ and 3′ flanking bases adjacent to each of substitution were integrated, thus 16 substitution trinucleotides were generated for each of these six subtypes. In total, 96 mutation motifs were generated to demonstrate all types of single base substitutions. We found that the most common substitution was ACG > ATG in all 26 CPs, followed by GCG > GTG and ATA > ACA substitutions (Fig. 2b). Moreover, GCG > GTG was the second frequent substitution in ACPs, but it was the fourth common in PCPs (Additional file 2: Fig. S5), indicating the slight difference of mutation pattern between two types of CPs.

Mutation signatures of our study cohort were analyzed with deconstructSigs. Because COSMIC signatures 1/5/6/11/14 and signatures 1/5/8 were previously identified in adult and pediatric brain cancers, respectively [30], these signatures were chosen to calculate their contributions to each patient. As shown in Fig. 2c, these signatures except for signature 14 contributed a lot to the mutational process of CPs, while signature 5 exhibited the most contribution (mean contribution: 65.23%, range 24.09–83.91%). Signature 5 is dominated by C:G > T:A and T:A > C:G mutations and also observed in other cancer types such as low grade glioma and lung cancer [30]. Age-related signature 1 was also found in 22 CPs (mean contribution: 17.94%) and indeed associated with CP patients’ age at diagnosis (Fig. 2d, *p* value = 0.0009979, r = 0.72 by Pearson’s correlation test). The recurrence of signature 1 in CP genomes suggests that this tumor might have existed for a long time of periods before diagnosis. In addition, we also analyzed the contributions of other 24 signatures with deconstructSigs, and found that there was scarcely contribution of other signatures in our CP cohort.

### Somatic mutations in protein-coding region

**Fig. 3:**
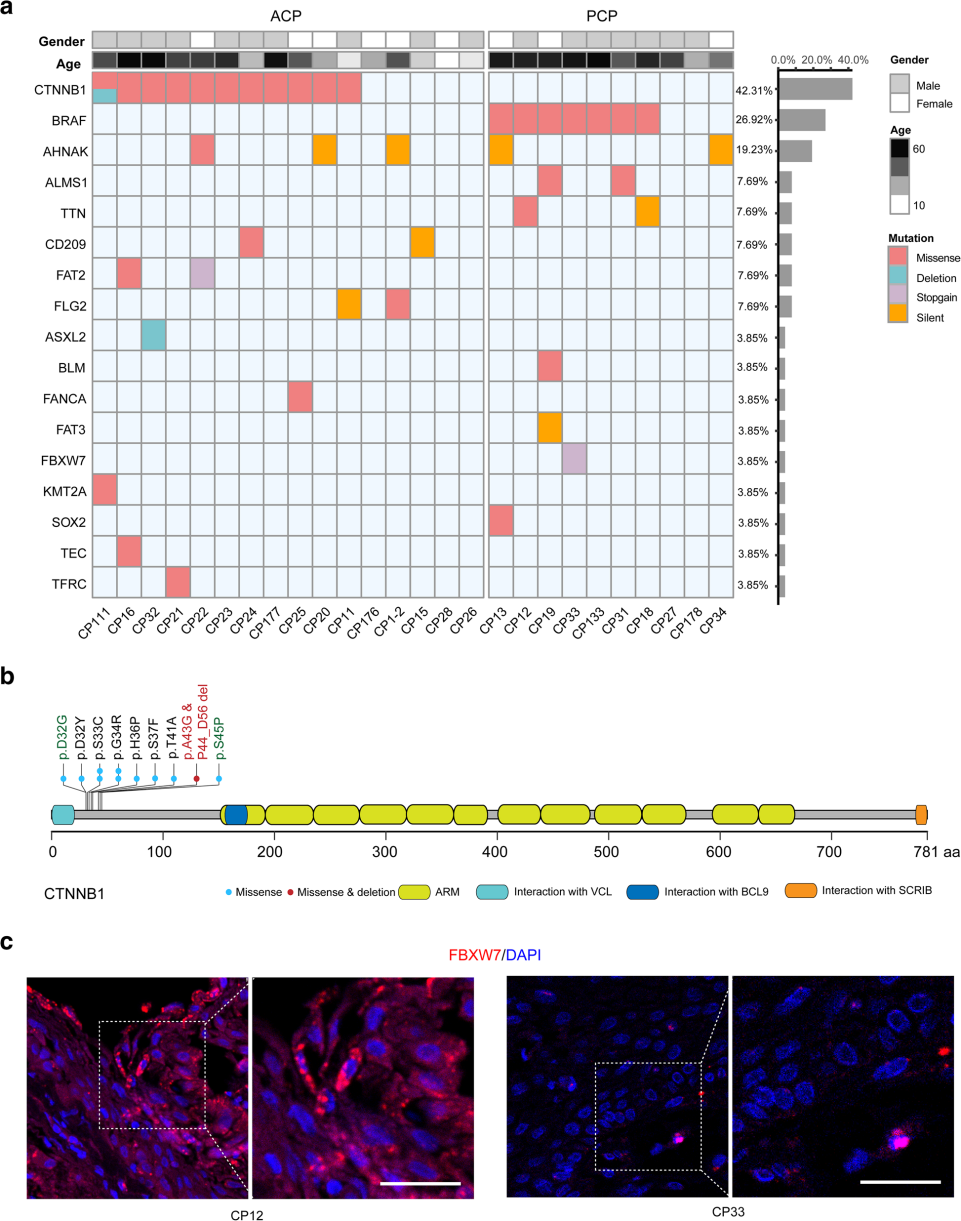
Repertoire of somatic alterations in coding regions of CP samples. (**a**) Age, gender, histological type (up) and somatic alterations in coding regions identified in 26 CPs subjected to WGS. The effects of the somatic alterations are color-coded according to the legend. Alterations that occurred in the same gene at least in two samples and/or listed in COSMIC Cancer Gene Census were shown. (**b**) Schematics for CTNNB1 indicating the location of the identified mutations, the mutation marked red was the novel mutation identified in CP111, and the mutation marked green were the mutations not identified in ref. [31]. (**c**) FISH images of FBXW7 shown for CP12 (FBXW7-WT) and CP33 (FBXW7 W425X), scale bars: 50 μm

Among 211 genes with mutations in protein-coding region, genes with mutations in at least two patients and/or listed in COSMIC Cancer Gene Census were shown in Fig. 3a (Additional file 2:Fig. S6; Additional file 5:Table S4). Consistent with previous reports [10], mutations in CTNNB1 and BRAF V600E were mutually exclusive in ACP and PCP, respectively (Fig. 3a). Eleven of 16 ACPs (68.75%) were identified to harbor mutations within the exon 3 of CTNNB1 gene (Fig. 3b, Additional file 2:Fig. S7 and Additional file 5:Table S4). Among these mutations, we identified a novel deletion (in this study later) and two missense mutations of CTNNB1 (p. D32G and p. S45P) which were not found in CPs before [10]. In PCPs, 7 of 10 patients (70.00%) were detected to have BRAF V600E mutation (Fig. 3a). Therefore, our results further consolidate the oncogenic roles of these two driver genes in ACP and PCP respectively.

Of the 164 missense mutations, 98 linked to 81 genes were predicted to be deleterious (Additional file 6:Table S5), implying their potential to promote tumorigenesis. For example, a deleterious missense mutation of CHD8 (Chromodomain helicase DNA binding protein 8, c.G1388A, p.R463Q) was identified in CP20 (ACP, female, age: 28). The chromatin remodeling enzyme CHD8 is a negative regulator of Wnt/β­catenin pathway [32] and this mutation may cause its function defective, thus leading to activation of Wnt signaling pathway to enhance tumorigenesis in CP20. Moreover, 38 predicted deleterious missense mutations were found to localize in important protein domains annotated in UniprotKB/Swiss-Prot (Additional file 6:Table S5, marked in red color), implying that such mutations may affect their protein functions. For instance, mutation in PIWIL1 (piwi like RNA-mediated gene silencing 1, c.A935G; p.Y312C) was embedded in its PAZ domain, an important domain for RNA binding to regulate gene expression. PIWIL1 was reported to participate in tumor progression via piRNA-dependent or -independent mechanisms [33, 34]. Therefore, it would be interesting to investigate the role of this PIWIL1 mutation in CP tumorigenesis in future.

Besides, nine genes were identified to harbor nonsense mutations (Additional file 7:Table S6). It is worth noting that a novel nonsense mutation within the WD40 domain of FBXW7 (F-box and WD repeat domain containing 7, c.G1274A, p.W425X) was identified in CP33 (PCP, male and age: 61) (Fig. 3a, Additional file 2:Fig. S6 and Fig. S8). Nonsense mutation introduces the premature termination codon (PTC) into the mRNAs that could be degraded by nonsense-mediated mRNA decay (NMD), an evolutionarily conserved cellular quality control mechanism to avoid producing truncated proteins [35]. To test this, we measured FBXW7 mRNA levels of patients by FISH technology. As shown in Fig. 3c, the mRNA level of FBXW7 was indeed lower in CP33 compared with that in CP12 (PCP, male and age: 58) with wilt-type of FBXW7, suggesting that such mutated mRNAs could be degraded by NMD. FBXW7 is a E3-ubiquitin ligase playing tumor suppressive roles by targeting some oncoproteins for ubiquitylation and proteasome degradation [36], so our results demonstrated another fate of tumor suppressor genes with nonsense mutations in tumor tissue. In addition, CP33 also harbors nonsense mutations in NEMF (Nuclear export mediator factor, c.760delA, p.I254X) and MYO18B (Myosin XVIIIb, c.C54443T, p.Q1815X), and the latter is another candidate tumor suppressor gene, while the interaction of these genes in CP33 needs to be further studied. Interestingly, BRAF V600E was also identified in this tumor sample (Fig. 3a). Aydin et al. found that FBXW7 inactivation in the mice with BRAF V600E mutation is consequential and sufficient to drive melanoma development [37], strongly supporting their cooperative functions during tumorigenesis. But the interaction of such mutations within these two genes in PCP needs to be further explored.

### CTNNB1-Mut increases β-catenin protein stability

**Fig. 4:**
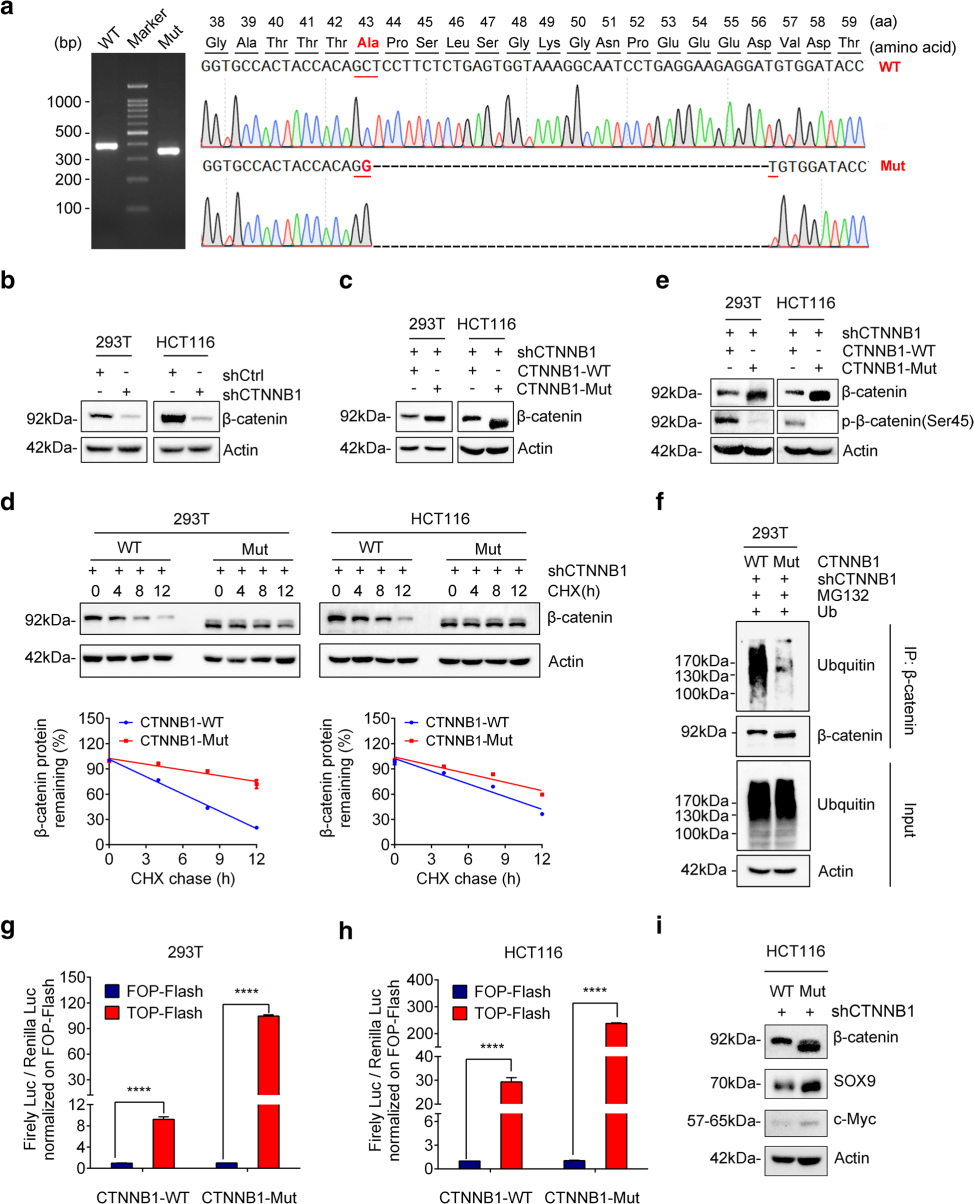
CTNNB1-Mut promotes Wnt/β-catenin signaling pathway via increasing the stability of β-catenin. (**a**) Agarose gel electrophoresis of PCR products generated by the cDNA of CP111 tissue (left panel) and schematic representation of CTNNB1 deletion mutants generated from ACP patient cells. WT: CTNNB1 wild type; Mut: CTNNB1 mutation type. (**b**) Knockdown efficiency of CTNNB1 shRNA (shCTNNB1) in both 293 T and HCT116 cells analyzed by western blotting. (**c**) Western blotting was performed to verify the expression of CTNNB1-WT/Mut 293 T/HCT116 cells. (**d**) CTNNB1-WT/Mut 293 T/HCT116 cells were cultured in the presence of CHX (50 μg/ml) for 0, 4, 8, 12 h, followed by immunoblotting (IB) using anti-β-catenin and actin antibodies. (**e**) CTNNB1-WT/Mut 293 T/HCT116 cells were treated with 50 nM Calyculin A for 30 min. The cell lysate was IB with anti-β-catenin and anti-β-catenin-phosphate-S45 antibodies. (**f**) Ubiquitin was transfected into 293 T cells which respectively infected with CTNNB1-WT or CTNNB1-Mut. After 48 h, cells were treated with 10 μM MG132 for 10 h. Cell lysates were subjected to IP with anti-β-catenin followed by IB with ubiquitin or anti-β-catenin antibody. (**g**-**h**) TOP-Flash reporter or FOP-Flash reporter was co-transfected with pRL-TK plasmids into CTNNB1-WT/Mut 293 T/HCT116 cells. Luciferase activity was measured with the Dual-Luciferase reporter assay, and relative luciferase activity was normalized to the FOP-Flash of the CTNNB1-WT group. Values are mean ± SD for triplicate samples. (**i**) Levels of SOX9 and c-Myc protein in HCT116-shCTNNB1 cells infected with CTNNB1-WT or CTNNB1-Mut were determined by Western blotting

As mentioned above, we identified a novel mutation (c.C127G, p.A43G; delP44_56D) in the exon 3 of CTNNB1 (CTNNB1-Mut) of the patient CP111 (ACP, male and age: 49) from our WGS results (Fig. 3b and Additional file 2:Fig. S7), which was further confirmed by Sanger sequencing (Fig. 4a). This mutation comprised of a deletion of 39 nucleotides and one missense substitution (C > G), thus causing an in-frame deletion of 13 amino-acid residuals and p.A43G change (Fig. 4a). Increasing studies indicate that somatic mutations within the exon 3 of CTNNB1 usually stabilize β-catenin protein and activate Wnt signaling pathway in cancers [8, 10, 11], but the biological function of this novel CTNNB1 mutations in ACP remains unknown.

Because it is difficult to prepare human ACP primary cells and there are no available ACP cultured cells, we firstly chose HEK293T (normal cells) and HCT116 cells (colorectal cancer cells) for our study. To prevent the effect of endogenous β-catenin on the mutant’s function, the endogenous β-catenin was knocked down by short hairpin RNA (shRNA) in HEK293T and HCT116 cells (Fig. 4b). Then stable cell lines expressing shRNA-resistant wild-type or mutated β-catenin were established in the β-catenin knockdown cells, and designated as CTNNB1-WT or CTNNB1-Mut cells, respectively. Intriguingly, we noticed that the β-catenin levels in CTNNB1-Mut cells were higher than those in CTNNB1-WT cells (Fig. 4c).

The increased β-catenin may be caused by the alteration of its mRNA or protein stability. To test this, we treated HEK293T or HCT116 stable cells with actinomycin D to terminate gene transcription for different times and then measured β-catenin mRNA levels. The results showed no significant difference of half-lives between WT and mutant β-catenin mRNAs (Additional file 2:Fig. S9), indicating that this mutation has no effect on its mRNA stability. Next, we treated cells with cycloheximide (CHX, a protein synthesis inhibitor) for different times and then performed Western blotting to check its protein stability. As shown in Fig. 4d, CTNNB1-Mut cells exhibited slower degradation of β-catenin than WT counterpart, suggesting that this novel mutation of CTNNB1 increased the stability of β-catenin.

Because this novel CTNNB1 mutation leads to loss of Ser45 residue phosphorylated by casein kinase I isoform-α (CK1α), so the Ser45 phosphorylation status of the mutant β-catenin was hardly detected (Fig. 4e). Given that Ser45 phosphorylation is critical for the polyubiquitination and subsequent proteasome degradation of β-catenin, we immunoprecipitated wild-type or mutant β-catenin to examine its ubiquitination status. As expected, the polyubiquitination of mutant β-catenin is dramatically lower that of wild-type one (Fig. 4f). Taken together, this novel deletion mutation of CTNNB1 increases β-catenin protein levels through impairing its degradation via ubiquitination-proteasome system.

### CTNNB1-Mut promotes Wnt/β-catenin signaling pathway

It has been reported that accumulated cytosolic β-catenin translocate to the nucleus where it promotes the transcription of Wnt target genes in association with T cell factor/lymphoid enhancer-binding factor (TCF/LEF) transcription factors [38]. To identify whether or not CTNNB1-Mut has the transcriptional activity, we conducted the TCF/LEF-responsive luciferase assay. The results showed either CTNNB1-Mut or CTNNB1-WT can stimulate the transcription of TOP-FLASH in both HEK293T (Fig. 4g) and HCT116 cells (Fig. 4h), indicating that this β-catenin mutant still keeps its transcriptional function. Because CTNNB1-Mut increased β-catenin expression through extending its protein half-life (Fig. 4d), the elevated activity of CTNNB1-Mut is much higher than that of CTNNB1-WT in both cells (Fig. 4g and h). In addition, we examined the expression levels of Wnt/β-catenin target genes such as c-Myc and SOX9, and found that the expression of c-Myc and SOX9 were significantly increased in CTNNB1-Mut cells when compared with CTNNB1-WT groups (Fig. 4i). Therefore, the CTNNB1 mutation identified in this study caused its protein stabilization to enhance expression of its downstream targets.

### CTNNB1-Mut promote ACP primary cell proliferation by activating Wnt signaling

**Fig. 5:**
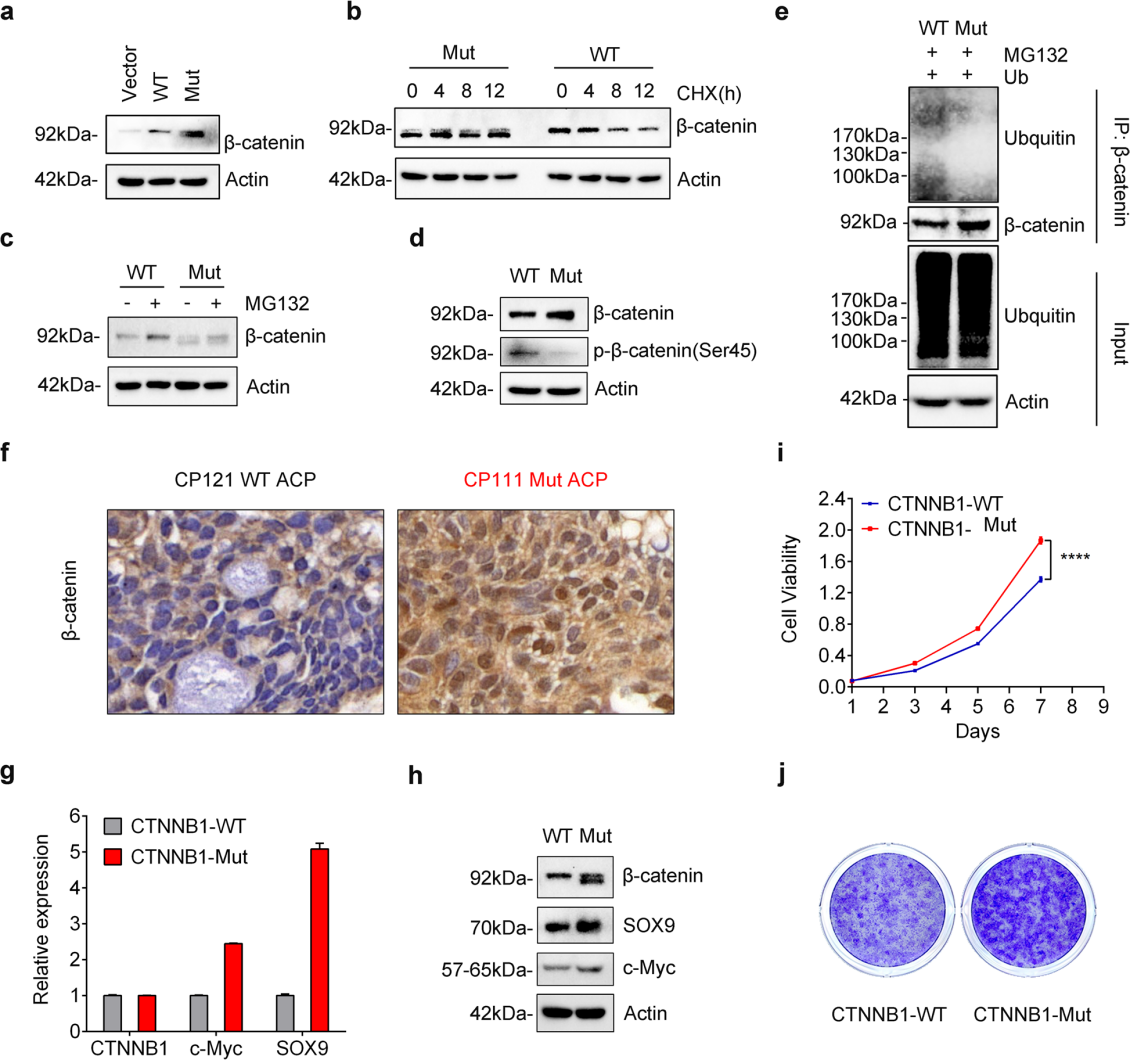
CTNNB1-Mut promote ACP primary cell proliferation by activating Wnt target genes. (**a**) Western blotting was performed to verify the overexpression of CTNNB1-WT and CTNNB1-Mut in ACP primary cells. (**b**) CTNNB1-WT/Mut ACP primary cells were treated with CHX (50 μg/ml) for 0, 4, 8, 12 h, followed by IB using anti-β-catenin and actin antibodies. (**c**) CTNNB1-WT/Mut ACP primary cells were treated with 10 μM MG132 for 10 h, and β-catenin expression levels were determined by western blotting. (**d**) CTNNB1-WT/Mut ACP primary cells were treated with 50 nM Calyculin A for 30 min. The cell lysate was IB with anti-β-catenin and anti-β-catenin-phosphate-S45 antibodies. (**e**) Ubiquitin was transfected into CTNNB1-WT/Mut ACP primary cells. After 48 h, cells were treated with 10 μM MG132 for 10 h. Cell lysates were subjected to IP with anti-β-catenin followed by IB with ubiquitin or anti-β-catenin antibody. (**f**) IHC of β-catenin in CP121 (CTNNB1 wild type in patient tissue), CP111 (CTNNB1 deletion mutants in patient sample), scale bars: 50 μm. (**g**-**h**) The mRNA (g) and protein (h) levels of SOX9 and c-Myc were examined in CTNNB1-WT/Mut ACP primary cells. (**i**-**j**) CCK8 assay (i) and colony-formation experiments (j) were performed to assess cell proliferation in the CTNNB1-WT/Mut in ACP primary cells. The results are shown as mean ± SD; *, *P* < 0.05; two-tailed Student *t*-test

To further explore the function of CTNNB1-Mut in the context of ACP, we prepared ACP primary cells and compared wild-type and mutant β-catenin expression. Same as the results in HEK293T and HCT116 cells, this CTNNB1 mutation leads to longer half-life and higher protein levels of β-catenin when compared to the wild-type (Fig. 5a and b). Notably, the protein abundance of wild-type β-catenin was increased in MG132-treated primary cells, while this phenomenon was not found for mutant β-catenin, indicating that the degradation pathway of the mutant was impaired (Fig. 5c). Meanwhile, we further verified that both Ser45 phosphorylation and ubiquitination level of mutant β-catenin dramatically reduced in ACP primary cells (Fig. 5d and e), consistent with our previous results (Fig. 4e and f). IHC analysis found that nuclear immunopositivity for β-catenin was obviously observed in ACP sample (CP111) with this CTNNB1 mutation, while it was almost absent in ACP sample (CP121) without CTNNB1 mutation (Fig. 5f). In addition, mutant β-catenin also activated the transcription of its well-known target genes (c-Myc and SOX9) in ACP primary cells, thus leading to the increase of their mRNA (Fig. 5g) and protein levels (Fig. 5h). To investigate the effects of CTNNB1-Mut on ACP tumorigenesis, we performed CCK8 and colony formation assays. As shown in Fig. 5i and j, mutant β-catenin markedly accelerated cell proliferation in ACP primary cells. In summary, these results proved that CTNNB1-Mut may promote ACP tumorigenesis through activating Wnt/β-catenin signaling pathway.

## Discussion

CPs are the histologically benign but clinically challenging neoplasms, including ACP and PCP subtypes. Recently, molecular characteristics of both subtypes of CPs were revealed by target sequencing or whole-exome sequencing [8–10]. But these sequencing technologies may miss key insight into some important genome region, and the complete spectrum of genetic changes of CPs remains elusive. Therefore, here we performed WGS to comprehensively understand genomic alternations of CPs. In this study, we analyzed 26 CPs (including 16 ACPs and 10 PCPs) and their matched normal blood samples. Our results showed that CP genomes exhibited much less somatic mutations than other malignant genomes. This could be explained by the benign histology of CPs. Moreover, we confirmed that CTNNB1 mutations are prevalent in ACPs (68.75%), and PCPs frequently harbor BRAF V600E (70.00%), and these mutations were mutually exclusive in ACP and PCP. Meanwhile, consistent with previous literatures [10, 13, 39], we also didn’t detect other recurrent mutations or genomic aberrations in either subtype, which could be due to the sequencing method used or the low proportion of tumour tissue within these tested samples [4]. Therefore, our results further consolidated the oncogenic roles of these two genes as driver genes in ACP and PCP, respectively.

Because we employed WGS to analyze the whole chromosomal sequences including introns and intergenic regions, our results identified more genetic alterations of CPs and provide deep insights of CP’s molecular signatures. For example, we found the relatively frequent mutations in AHNAK in our cohort (5/26) (Fig. 3a, Additional file 2:Fig. S6; Additional file 5:Table S4). AHNAK plays important roles in multiple signaling pathways such as TGFβ/Smad signaling [40], so dysregulation of its expression was reported to promote tumorigenesis [41–43]. In addition, AHNAK mutation was considered as a prognostic factor associated with poor survival of patients with different cancers [41–43]. Here we identified that CPs harbor five mutations within AHNAK gene, including 1 missense mutation and 4 silent mutations, implying their significance in CPs. Our results provide some open questions to be addressed: whether this missense mutation changes the function of AHNAK in CPs? Whether these mutations could serve as the prognostic marker for CPs?

To understand the potential effect of mutations on their functions, we used the bioinformatics tools to predict the deleterious mutations and then analyze their localizations within genes. Interestingly, we found that 38 predicted deleterious missense mutations localize in important domains of proteins, suggesting that such mutations may affect their protein functions (Additional file 6:Table S5, marked in red color). For example, mutations in TEC (Tec protein tyrosine kinase, c.A122G, p.Y41C) and ABR (Active breakpoint cluster region-related protein, c.C1079G, p.P360R) were embedded in PH (Pleckstrin homology) domain, a critical domain to bind phosphoinositides for regulation of intracellular signaling [44]. Tec kinase is an integral component of T cell signaling and controls early cell fate decisions of human pluripotent stem cells via regulation of fibroblast growth factor-2 secretion [45]. ABR is an important regulator of neuronal development [46]. Presumably, mutations in TEC and ABR may affect its binding with phosphoinositides and downstream signaling cascade. Thus, our results provided abundant deleterious missense mutations to be experimentally validated for their roles in CP tumorigenesis, possibly elucidating the molecular mechanisms of these genes during CP progression. In this study, we focused on the novel mutation in the exon 3 of CTNNB1 gene.

Somatic mutations in CTNNB1 were reported to stabilize β-catenin protein that translocate to the nucleus to activate Wnt signaling pathway in several cancers [1, 38]. Among these mutations, the most common are point mutations within the exon 3 of CTNNB1 gene, occurring at serine (S)/threonine (T) residues (S33, S37, T41, and S45) in the N-terminal region of β-catenin that is essential for its ubiquitination and subsequent proteasome degradation [38]. In this study, we identified a novel mutation within the exon 3 of CTNNB1 (CTNNB1-Mut) from ACP, which comprised of a deletion of 39 nucleotides and one missense substitution (C > G), thus causing an in-frame deletion of 13 amino-acid residuals and p.A43G change (Fig. 4a). Notably, this mutant β-catenin lacks the classical phosphorylation residue S45. No matter in HEK293T, HCT116 and ACP primary cells, CTNNB1-Mut exhibited higher protein levels than the wild-type (Fig. 4c and Fig. 5a). Moreover, IHC analysis also revealed that this CTNNB1 mutation elevated β-catenin expression in the patient’s sample (CP111) (Fig. 5f). The increase of mutant β-catenin was caused by its less polyubiquitination (Fig. 4f and Fig. 5e) and sequent proteasome degradation, thus extending the half-life of mutant protein (Fig. 4d and Fig. 5b). It was reported that β-catenin with only S45 point mutation was not entirely resistant to proteolytic degradation in colorectal cancer cells [47], so this mutant β-catenin identified in CP could have better protein stability than the S45 single point mutation. As expected, wild-type β-catenin was degraded through the ubiquitin-proteasome pathway in ACP primary cells, evidenced by the increase of its protein level after MG132 treatment (Fig. 5c). However, MG132 treatment didn’t cause obvious accumulation of mutant β-catenin protein (Fig. 5c), suggesting the degradation of mutant β-catenin is not mediated by proteasomes. The underlying mechanism of mutant β-catenin turnover needs further investigation. Because CTNNB1-Mut deletes 39 nucleotides encoding 13 amino acids, it is not clear whether the mutant β-catenin altered its protein conformation, protein-protein interaction or post-translational modification.

Even though mutations in CTNNB1 gene elevated β-catenin levels, whether this mutant β-catenin identified in ACP is functional remains unknown. To address this, we firstly expressed CTNNB1-Mut in HEK293T or HCT116 cells and then performed TCF/LEF-responsive luciferase assays. The results demonstrated that mutant β-catenin promoted higher luciferase activity than the wild-type (Fig. 4g and h). Moreover, mutant β-catenin dramatically enhanced the expression of Wnt target genes (c-Myc and SOX9) in both HCT116 (Fig. 4i) and ACP primary cells (Fig. 5g and h). Both c-Myc and SOX9 were reported to play essential roles in normal cells and frequently dysregulated in human cancers [48–50]. For example, c-Myc and SOX9 were remarkably upregulated in the colorectal cancer and can accelerate cell proliferation [48, 51]. In ACP primary cells, mutant β-catenin also promoted faster cell proliferation than the wild-type (Fig. 5i and j). In uterine leiomyomas, CTNNB1 mutant cell sends the paracrine mitogenic signal to adjacent tumor cells and facilitates tumor cell proliferation [52]. Therefore, ACP cell clusters with mutant β-catenin may also activate the Wnt signaling pathway and affect other surrounding tumor growth in a same manner. Taken together, our data demonstrated that accumulated β-catenin mutants can facilitate the transcription factors of TCF/LEF family to activate the expression of Wnt target genes, thus accelerating cell proliferation in ACP primary cells.

## Conclusions

Whole genome landscapes of both ACPs and PCPs were revealed for the first time, and we found that CP genome exhibited less somatic mutations than other malignant tumors. Moreover, we confirmed that mutations in CTNNB1 (68.75% of ACPs) and BRAF V600E (70.00% of PCPs) were mutually exclusive in ACP and PCP, respectively. Intriguingly, a novel mutation in CTNNB1 gene was identified, which compromised both a transversion and in-frame deletion, and this mutation conferred increased stability of β-catenin protein through inhibiting its ubiquitination, thus activating Wnt/β-catenin signaling pathway.

## Supplementary Information

**Additional file 1: Table S1.** Sample information and quality of WGS data.

**Additional file 2: Figs. S1-S9.** Supplementary figures and their corresponding figure legends.

**Additional file 3: Table S2.** The detailed information of variants spectrum for each patient.

**Additional file 4: Table S3.** Somatic structural variations called in each patient.

**Additional file 5: Table S4.** Exonic mutations in genes listed in Fig. 3a.

**Additional file 6: Table S5.** Deleterious missense mutations identified in CP.

**Additional file 7: Table S6.** Nonsense mutations identified in CP.

## Data Availability

The datasets used in this study have been submitted to the Genome Sequence Archive (GSA) database, China National Center for Bioinformation under accession number (we are submitting the data and will provide the number later).

## Abbreviations

CP: Craniopharyngioma
ACP: Adamantinomatous-type craniopharyngioma
PCP: Papillary-type craniopharyngioma
WGS: Whole genome sequencing
CTNNB1: Catenin Beta 1
BRAF: B-Raf Proto-Oncogene, Serine/Threonine Kinase
GTR: Gross-total resection
STR: Sub-total resection
MAPK pathway: The mitogen-activated protein kinase pathway
SNVs: Single nucleotide variants
InDels: Insertions and Deletions
TMB: Tumor mutational burden
SV: Structural Variant
COSMIC: Catalogue of Somatic Mutations in Cancer
FBXW7: F-box and WD repeat domain containing 7
PTC: Premature termination codon
NMD pathway: Nonsense-mediated mRNA decay pathway
CDH8: Chromodomain helicase DNA binding protein 8
SOX9: SRY-box 9
IP: Immunoprecipitation
IB: Immunoblotting
IHC: Immunohistochemical staining
CCK-8: Cell viability was measured using Cell Counting Kit-8
TO: A new type of biological tissue transparent agent

## References

[1] Müller HL, Merchant TE, Warmuth-Metz M, Martinez-Barbera JP, Puget S (2019). Craniopharyngioma. Nat Rev Dis Primers.

[2] Momin AA, Recinos MA, Cioffi G, Patil N, Soni P, Almeida JP (2021). Descriptive epidemiology of craniopharyngiomas in the United States. Pituitary..

[3] Muller HL, Merchant TE, Puget S, Martinez-Barbera JP (2017). New outlook on the diagnosis, treatment and follow-up of childhood-onset craniopharyngioma. Nat Rev Endocrinol.

[4] Schoenfeld A, Pekmezci M, Barnes MJ, Tihan T, Gupta N, Lamborn KR (2012). The superiority of conservative resection and adjuvant radiation for craniopharyngiomas. J Neuro-Oncol.

[5] Barkhoudarian G, Laws ER (2013). Craniopharyngioma: history. Pituitary.

[6] Martinez-Barbera JP, Buslei R (2015). Adamantinomatous craniopharyngioma: pathology, molecular genetics and mouse models. J Pediatr Endocrinol Metab.

[7] Wang Y, Deng J, Wang L, Zhou T, Yang J, Tian Z (2020). Expression and clinical significance of PD-L1, B7-H3, B7-H4 and VISTA in craniopharyngioma. J Immunother Cancer.

[8] Buslei R, Nolde M, Hofmann B, Meissner S, Eyupoglu IY, Siebzehnrubl F (2005). Common mutations of beta-catenin in adamantinomatous craniopharyngiomas but not in other tumours originating from the sellar region. Acta Neuropathol.

[9] Campanini ML, Colli LM, Paixao BM, Cabral TP, Amaral FC, Machado HR (2010). CTNNB1 gene mutations, pituitary transcription factors, and microRNA expression involvement in the pathogenesis of adamantinomatous craniopharyngiomas. Horm Cancer.

[10] Brastianos PK, Taylor-Weiner A, Manley PE, Jones RT, Dias-Santagata D, Thorner AR (2014). Exome sequencing identifies BRAF mutations in papillary craniopharyngiomas. Nat Genet.

[11] Sekine S, Shibata T, Kokubu A, Morishita Y, Noguchi M, Nakanishi Y (2002). Craniopharyngiomas of adamantinomatous type harbor beta-catenin gene mutations. Am J Pathol.

[12] Kato K, Nakatani Y, Kanno H, Inayama Y, Ijiri R, Nagahara N (2004). Possible linkage between specific histological structures and aberrant reactivation of the Wnt pathway in adamantinomatous craniopharyngioma. J Pathol.

[13] Goschzik T, Gessi M, Dreschmann V, Gebhardt U, Wang L, Yamaguchi S (2017). Genomic alterations of adamantinomatous and papillary craniopharyngioma. J Neuropathol Exp Neurol.

[14] Li H, Durbin R (2009). Fast and accurate short read alignment with burrows-wheeler transform. Bioinformatics..

[15] McKenna A, Hanna M, Banks E, Sivachenko A, Cibulskis K, Kernytsky A (2010). The genome analysis toolkit: a MapReduce framework for analyzing next-generation DNA sequencing data. Genome Res.

[16] Cibulskis K, Lawrence MS, Carter SL, Sivachenko A, Jaffe D, Sougnez C (2013). Sensitive detection of somatic point mutations in impure and heterogeneous cancer samples. Nat Biotechnol.

[17] Kim S, Scheffler K, Halpern AL, Bekritsky MA, Noh E, Kallberg M (2018). Fast and accurate calling of germline and somatic variants. Nat Methods.

[18] Koboldt DC, Zhang Q, Larson DE, Shen D, McLellan MD, Lin L (2012). VarScan 2: somatic mutation and copy number alteration discovery in cancer by exome sequencing. Genome Res.

[19] Lee JJ, Park S, Park H, Kim S, Lee J, Lee J (2019). Tracing oncogene rearrangements in the mutational history of lung adenocarcinoma. Cell..

[20] Favero F, Joshi T, Marquard AM, Birkbak NJ, Krzystanek M, Li Q (2015). Sequenza: allele-specific copy number and mutation profiles from tumor sequencing data. Ann Oncol.

[21] Rosenthal R, McGranahan N, Herrero J, Taylor BS, Swanton C (2016). DeconstructSigs: delineating mutational processes in single tumors distinguishes DNA repair deficiencies and patterns of carcinoma evolution. Genome Biol.

[22] Chalmers ZR, Connelly CF, Fabrizio D, Gay L, Ali SM, Ennis R (2017). Analysis of 100,000 human cancer genomes reveals the landscape of tumor mutational burden. Genome Med.

[23] Wang L, Pan S, Zhu B, Yu Z, Wang W (2021). Comprehensive analysis of tumour mutational burden and its clinical significance in prostate cancer. BMC Urol.

[24] Jackson SP, Bartek J (2009). The DNA-damage response in human biology and disease. Nature..

[25] Lawrence MS, Stojanov P, Polak P, Kryukov GV, Cibulskis K, Sivachenko A (2013). Mutational heterogeneity in cancer and the search for new cancer-associated genes. Nature..

[26] Cesare AJ, Kaul Z, Cohen SB, Napier CE, Pickett HA, Neumann AA (2009). Spontaneous occurrence of telomeric DNA damage response in the absence of chromosome fusions. Nat Struct Mol Biol.

[27] Pfeifer GP, Besaratinia A (2009). Mutational spectra of human cancer. Hum Genet.

[28] Nik-Zainal S, Davies H, Staaf J, Ramakrishna M, Glodzik D, Zou X (2016). Landscape of somatic mutations in 560 breast cancer whole-genome sequences. Nature..

[29] Harris RS (2013). Cancer mutation signatures, DNA damage mechanisms, and potential clinical implications. Genome Med..

[30] Alexandrov LB, Nik-Zainal S, Wedge DC, Aparicio SA, Behjati S, Biankin AV (2013). Signatures of mutational processes in human cancer. Nature..

[31] Holsken A, Buchfelder M, Fahlbusch R, Blumcke I, Buslei R (2010). Tumour cell migration in adamantinomatous craniopharyngiomas is promoted by activated Wnt-signalling. Acta Neuropathol.

[32] Nishiyama M, Skoultchi AI, Nakayama KI (2012). Histone H1 recruitment by CHD8 is essential for suppression of the Wnt-beta-catenin signaling pathway. Mol Cell Biol.

[33] Shi S, Yang ZZ, Liu S, Yang F, Lin H (2020). PIWIL1 promotes gastric cancer via a piRNA-independent mechanism. Proc Natl Acad Sci U S A.

[34] Xie J, Xing S, Shen BY, Chen HT, Sun B, Wang ZT (2021). PIWIL1 interacting RNA piR-017061 inhibits pancreatic cancer growth via regulating EFNA5. Hum Cell.

[35] Kurosaki T, Popp MW, Maquat LE (2019). Quality and quantity control of gene expression by nonsense-mediated mRNA decay. Nat Rev Mol Cell Biol.

[36] Welcker M, Clurman BE (2008). FBW7 ubiquitin ligase: a tumour suppressor at the crossroads of cell division, growth and differentiation. Nat Rev Cancer.

[37] Aydin IT, Abbate F, Rajan GS, Badal B, Aifantis I, Desman G, Celebi JT (2017). FBXW7 inactivation in a Braf(V600E) -driven mouse model leads to melanoma development. Pigment Cell Melanoma Res.

[38] Bugter JM, Fenderico N, Maurice MM (2014). Mutations and mechanisms of WNT pathway tumour suppressors in cancer. Nat rev Cancer. 2021;21(1):5-21.39. Lee IH, Sohn M, Lim HJ, Yoon S, Oh H, shin S, et al. AHNAK functions as a tumor suppressor via modulation of TGFbeta/Smad signaling pathway. Oncogene..

[39] Apps JR, Carreno G, Gonzalez-Meljem JM, Haston S, Guiho R, Cooper JE (2018). Tumour compartment transcriptomics demonstrates the activation of inflammatory and odontogenic programmes in human adamantinomatous craniopharyngioma and identifies the MAPK/ERK pathway as a novel therapeutic target. Acta Neuropathol.

[40] Silva TA, Smuczek B, Valadao IC, Dzik LM, Iglesia RP, Cruz MC (2016). AHNAK enables mammary carcinoma cells to produce extracellular vesicles that increase neighboring fibroblast cell motility. Oncotarget..

[41] Sheppard HM, Feisst V, Chen J, Print C, Dunbar PR (2016). AHNAK is downregulated in melanoma, predicts poor outcome, and may be required for the expression of functional cadherin-1. Melanoma Res.

[42] Chen B, Wang J, Dai D, Zhou Q, Guo X, Tian Z (2017). AHNAK suppresses tumour proliferation and invasion by targeting multiple pathways in triple-negative breast cancer. J Exp Clin Cancer Res.

[43] Dumitru CA, Bankfalvi A, Gu X, Zeidler R, Brandau S, Lang S (2013). AHNAK and inflammatory markers predict poor survival in laryngeal carcinoma. PLoS One.

[44] Singh N, Reyes-Ordoñez A, Compagnone MA, Moreno JF, Leslie BJ, Ha T (2021). Redefining the specificity of phosphoinositide-binding by human PH domain-containing proteins. Nat Commun.

[45] Vanova T, Konecna Z, Zbonakova Z, Venuta GL, Zoufalova K, Jelinkova S (2017). Tyrosine kinase expressed in hepatocellular carcinoma, TEC, controls pluripotency and early cell fate decisions of human pluripotent stem cells via regulation of fibroblast growth factor-2 secretion. Stem Cells.

[46] Oh D, Han S, Seo J, Lee JR, Choi J, Groffen J (2010). Regulation of synaptic Rac1 activity, long-term potentiation maintenance, and learning and memory by BCR and ABR Rac GTPase-activating proteins. J Neurosci.

[47] Wang ZH, Vogelstein B, Kinzler KW (2003). Phosphorylation of beta-catenin at S33, S37, or T41 can occur in the absence of phosphorylation at T45 in colon cancer cells. Cancer Res.

[48] He TC, Sparks AB, Rago C, Hermeking H, Zawel L, da Costa LT (1998). Identification of c-MYC as a target of the APC pathway. Science..

[49] Lourenco C, Resetca D, Redel C, Lin P, MacDonald AS, Ciaccio R (2021). MYC protein interactors in gene transcription and cancer. Nat Rev Cancer.

[50] Panda M, Tripathi SK, Biswal BK (2021). SOX9: an emerging driving factor from cancer progression to drug resistance. Biochim Biophys Acta Rev Cancer.

[51] Zhou TC, Wu LL, Ma N, Tang FX, Yu ZM, Jiang ZP (2020). SOX9-activated FARSA-AS1 predetermines cell growth, stemness, and metastasis in colorectal cancer through upregulating FARSA and SOX9. Cell Death Dis.

[52] Ono M, Yin P, Navarro A, Moravek MB, JSt C, Druschitz SA (2013). Paracrine activation of WNT/beta-catenin pathway in uterine leiomyoma stem cells promotes tumor growth. Proc Natl Acad Sci U S A.

