# Supplementary material for: Characterization of novel CTNNB1 mutation in Craniopharyngioma by whole-genome sequencing
Source: Mol Cancer. 2021 Dec 18;20:168. doi: 10.1186/s12943-021-01468-7 (PMC8684236; doi:10.1186/s12943-021-01468-7)
Supplement: Supplementary file 2 — Additional file 2: Figs. S1-S9. Supplementary figures and their corresponding figure legends. [file 12943_2021_1468_MOESM2_ESM.pdf]

Supplementary Figures

Fig S1. The age and gender distribution of ACP and PCP group.

Fig S2. The correlation between somatic mutation number and age.

Fig S3. Structure alterations called in each patient.

Fig S4. Kataegis analysis of somatic mutations (SNVs and InDels) in each patient.

Fig S5. The proportion of 96 different extending possible base-pair substitutions of all SNVs identified in ACPs and 10 PCPs.

Fig S6. Schematics for genes listed in Fig. 3a indicating the location of the identified mutations.

Fig S7. IGV graphic report for the alignment of deletion in CTNNB1 CP111 tumor and normal blood sample.

Fig S8. IGV graphic report for the alignment of nonsense mutation of W425X in FBXW7 of CP22 tumor and normal blood sample.

Fig S9. CTNNB1 mRNA stability in CTNNB1-WT/Mut 293T/HCT116 cells.

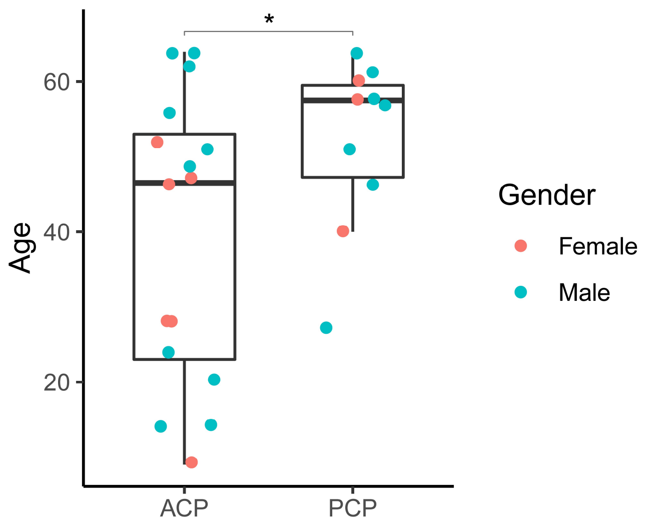

Fig S1. The age and gender distribution of ACP and PCP group.

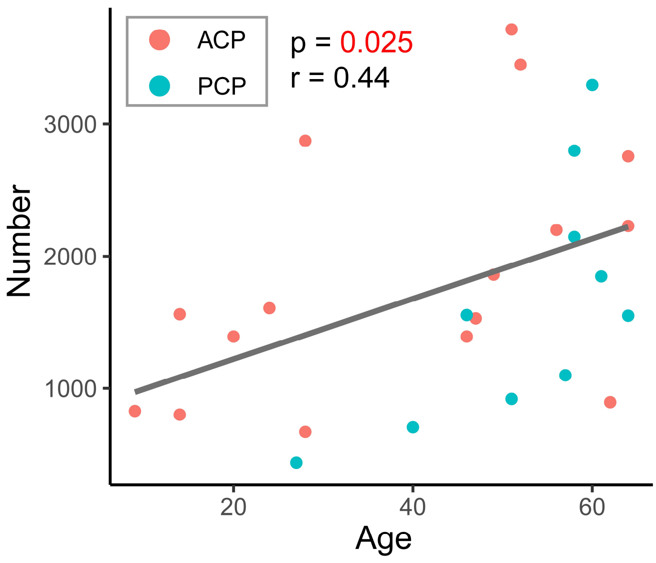

Fig S2. The correlation between somatic mutation number and age.

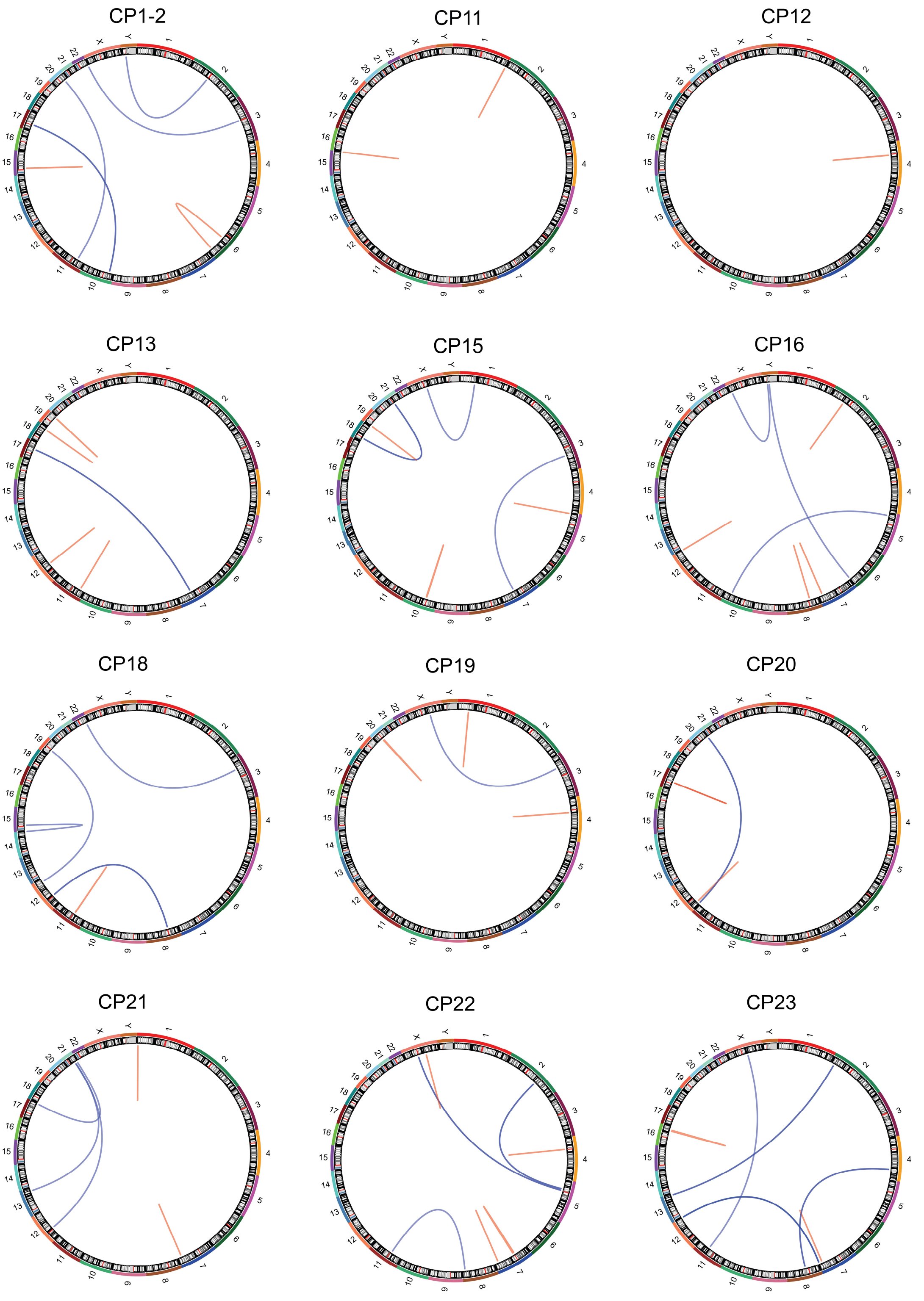
CP1-2
CP11
CP12
CP13
CP15
CP16
CP18
CP19
CP20
CP21
CP22
CP23

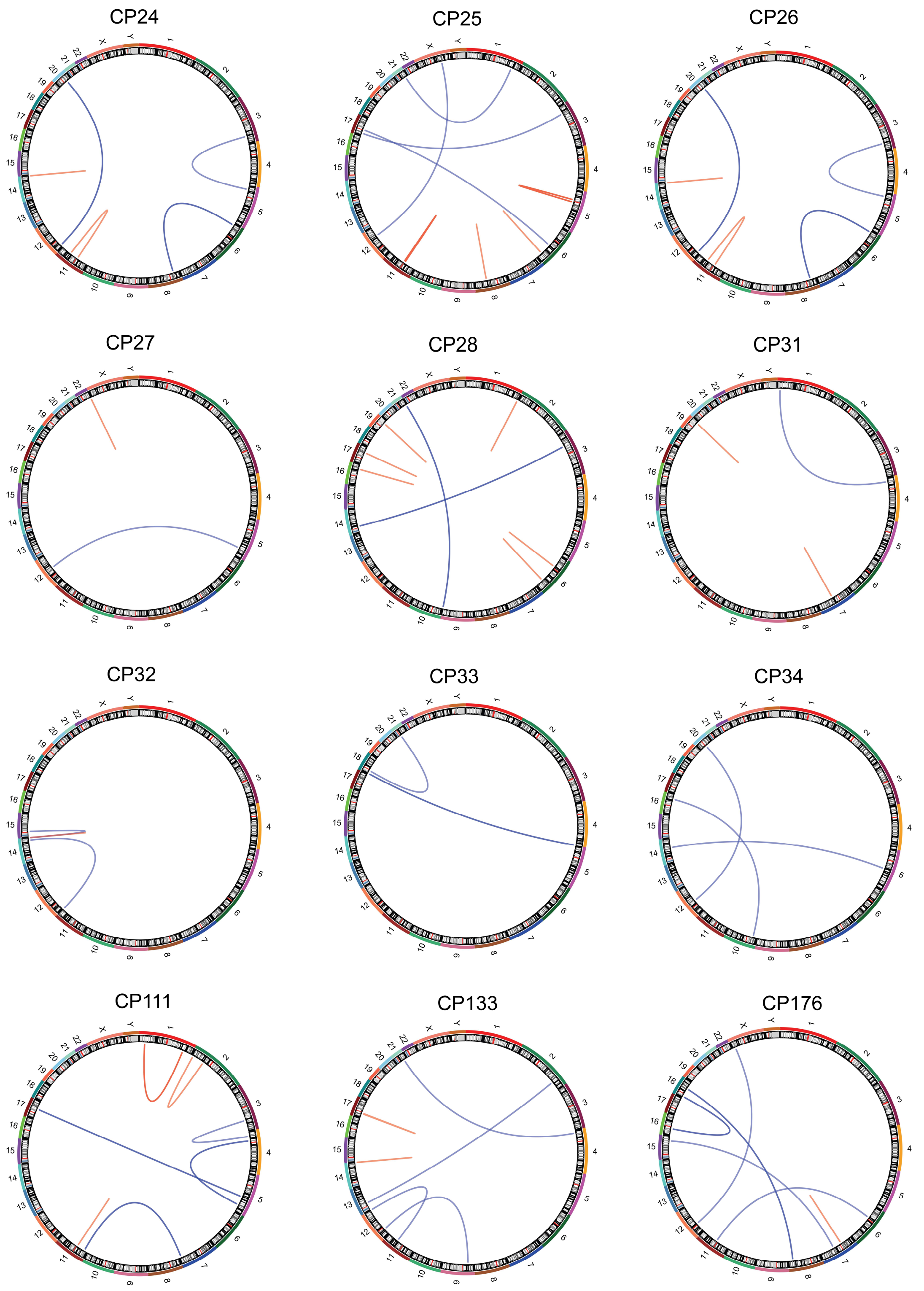
CP24
CP25
CP26
CP27
CP28
CP31
CP32
CP33
CP34
CP111
CP133
CP176

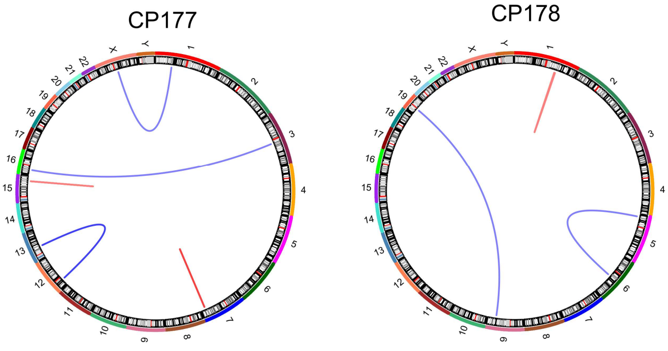

Fig S3. Structure alterations called in each patient.

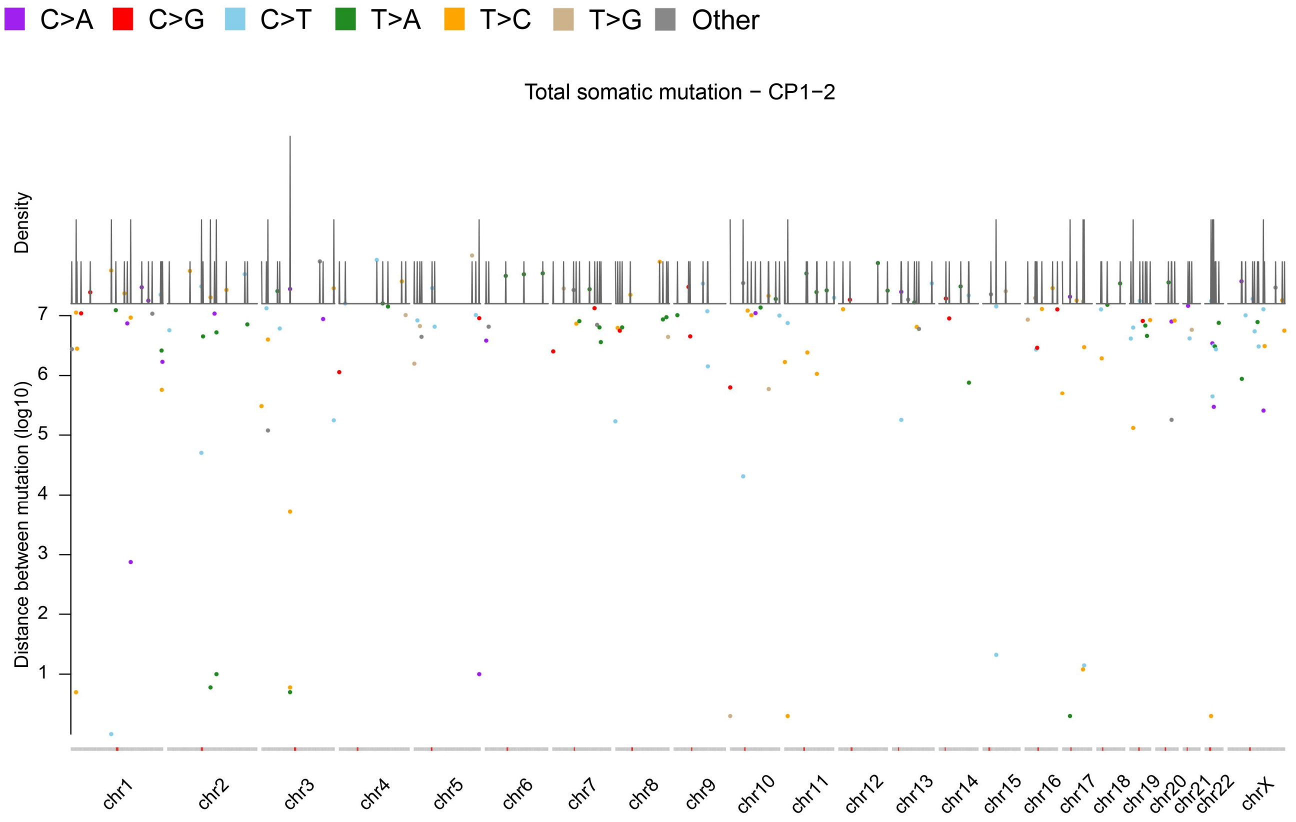

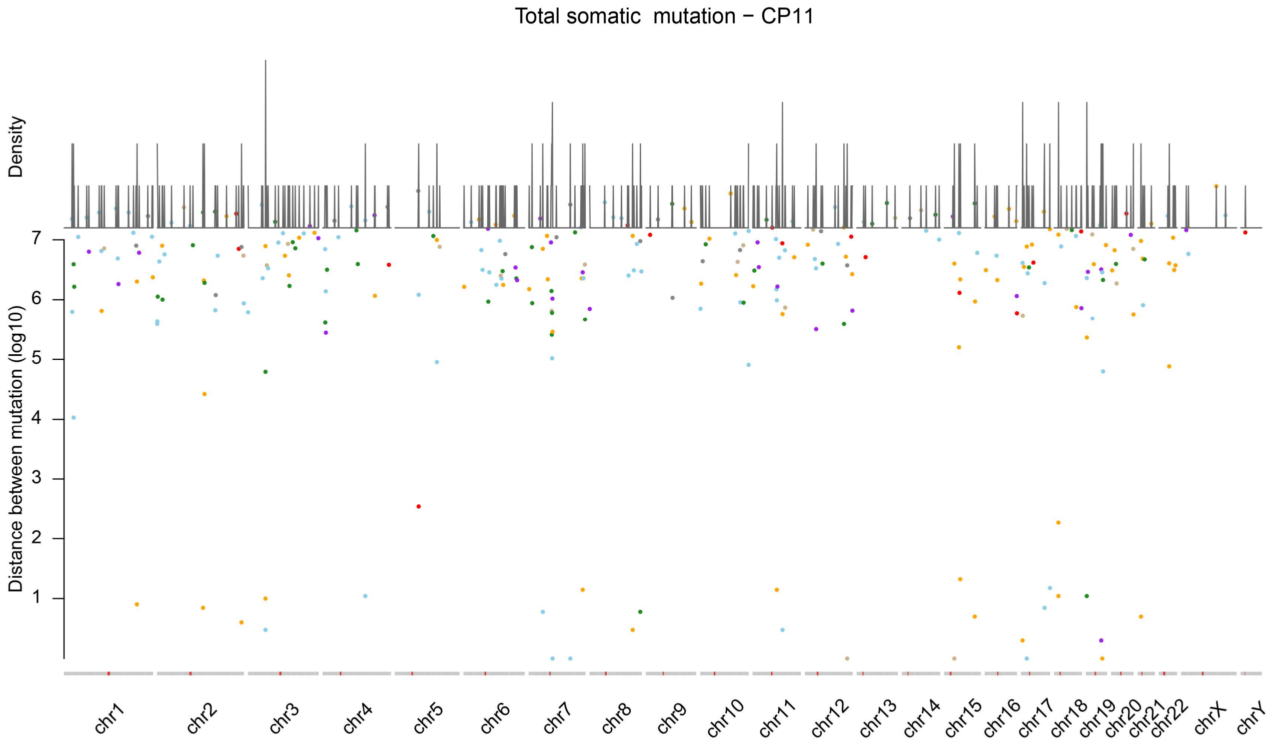
Total somatic mutation − CP11
Density
Distance between mutation (log10)
7
6
5
4
3
2
1
chr1
chr2
chr3
chr4
chr5
chr6
chr7
chr8
chr9
chr10
chr11
chr12
chr13
chr14
chr15
chr16
chr17
chr18
chr19
chr20
chr21
chr22
chrX
chrY

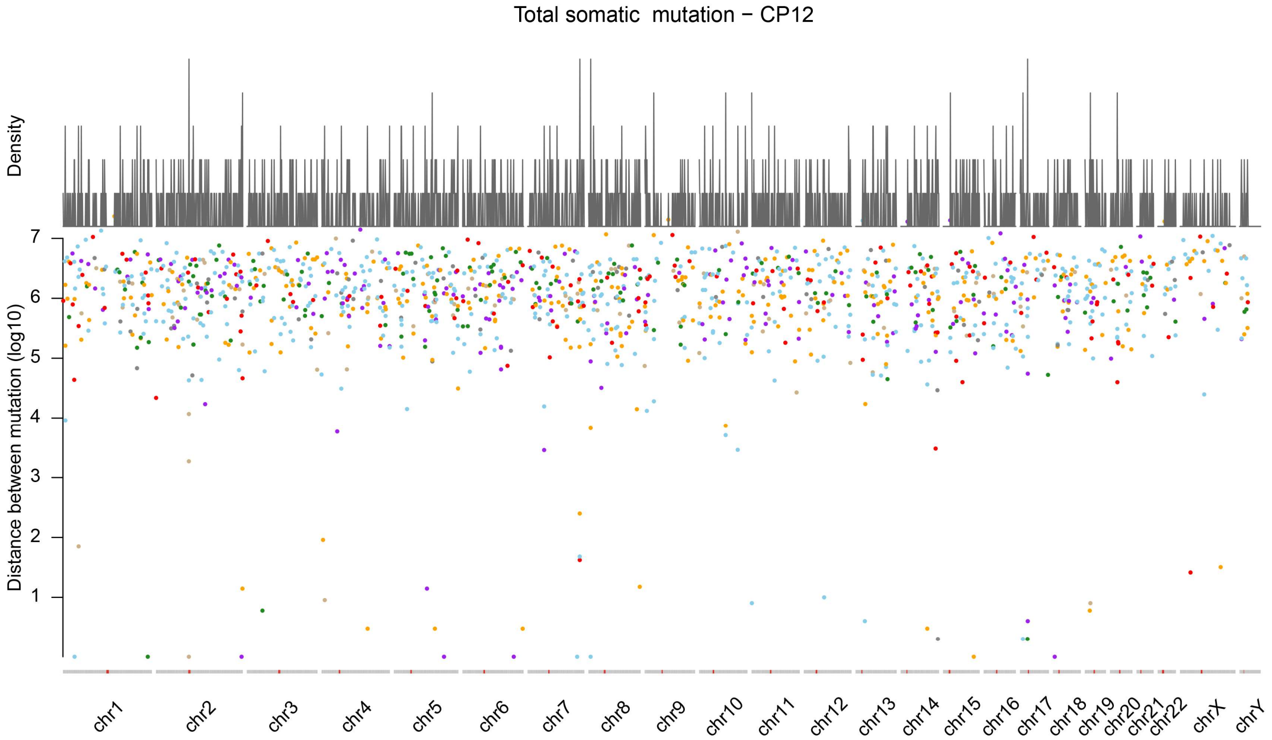
Total somatic mutation − CP12
Density
Distance between mutation (log10)
7
6
5
4
3
2
1
chr1
chr2
chr3
chr4
chr5
chr6
chr7
chr8
chr9
chr10
chr11
chr12
chr13
chr14
chr15
chr16
chr17
chr18
chr19
chr20
chr21
chr22
chrX
chrY

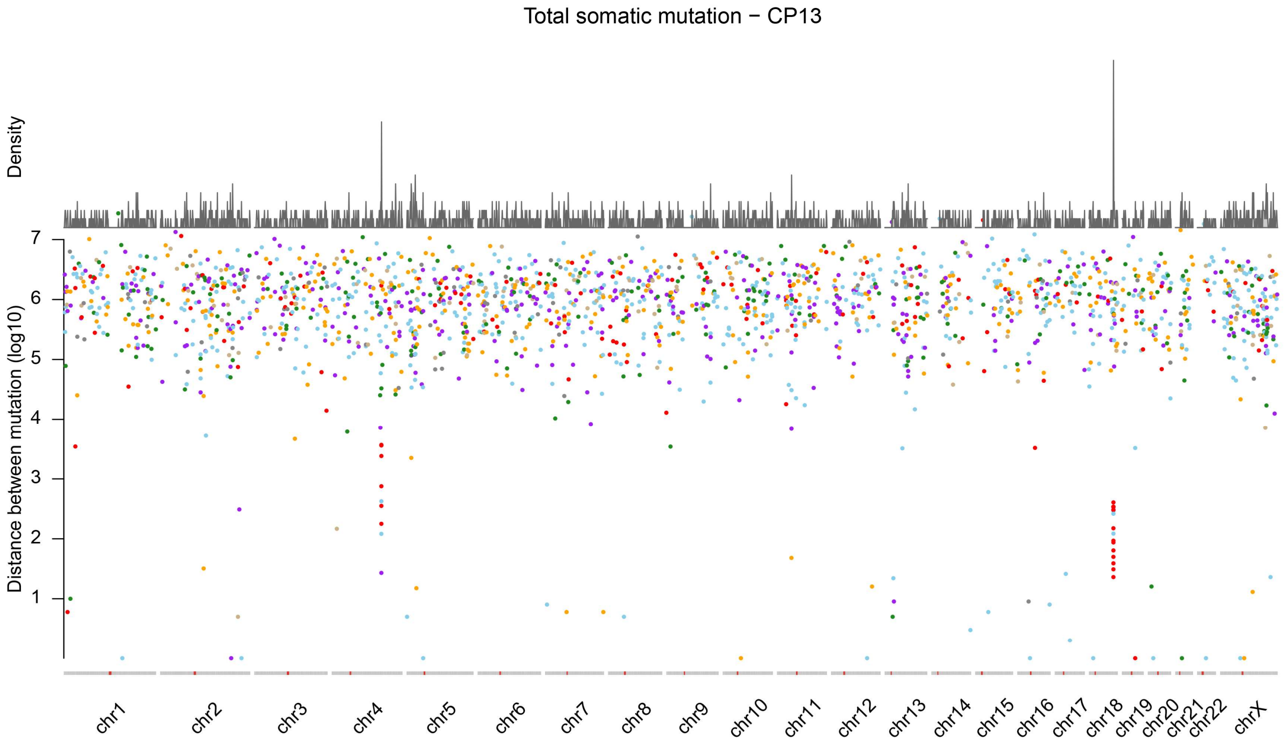
Total somatic mutation − CP13
Density
Distance between mutation (log10)
7
6
5
4
3
2
1
chr1
chr2
chr3
chr4
chr5
chr6
chr7
chr8
chr9
chr10
chr11
chr12
chr13
chr14
chr15
chr16
chr17
chr18
chr19
chr20
chr21
chr22
chrX

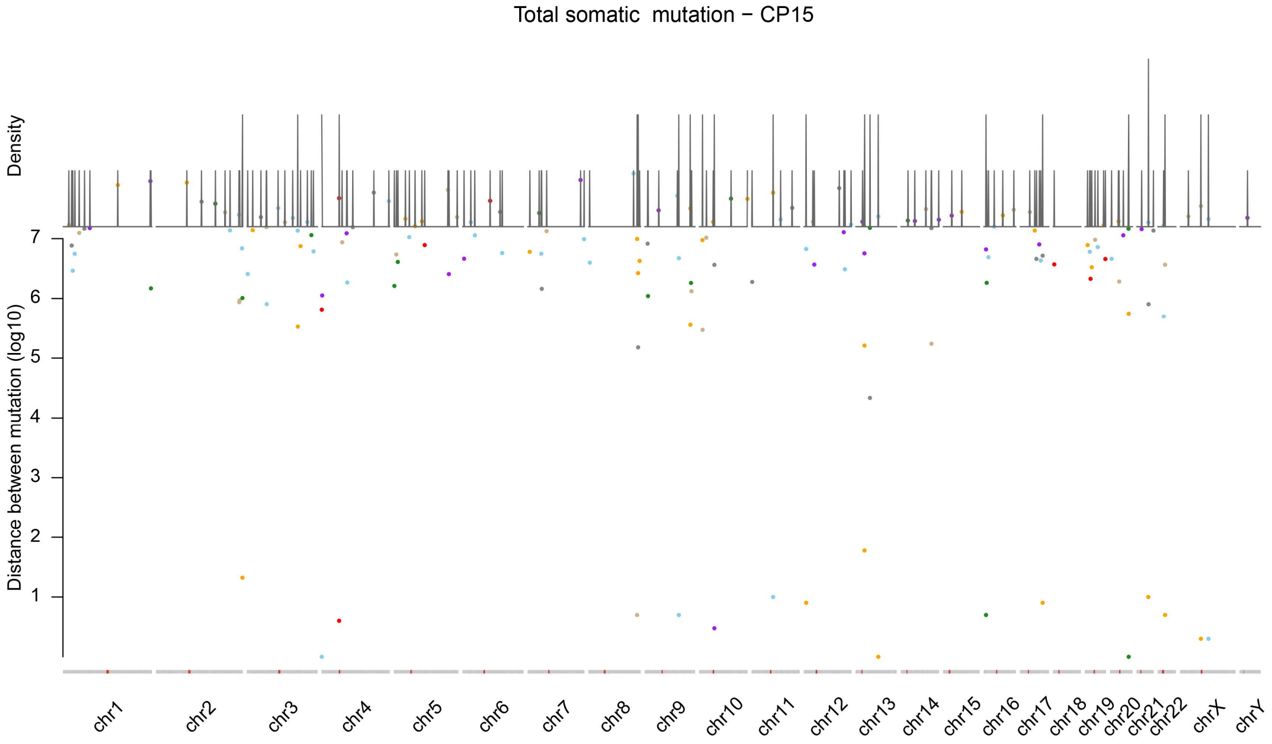
Total somatic mutation − CP15
Density
Distance between mutation (log10)
7
6
5
4
3
2
1
chr1
chr2
chr3
chr4
chr5
chr6
chr7
chr8
chr9
chr10
chr11
chr12
chr13
chr14
chr15
chr16
chr17
chr18
chr19
chr20
chr21
chr22
chrX
chrY

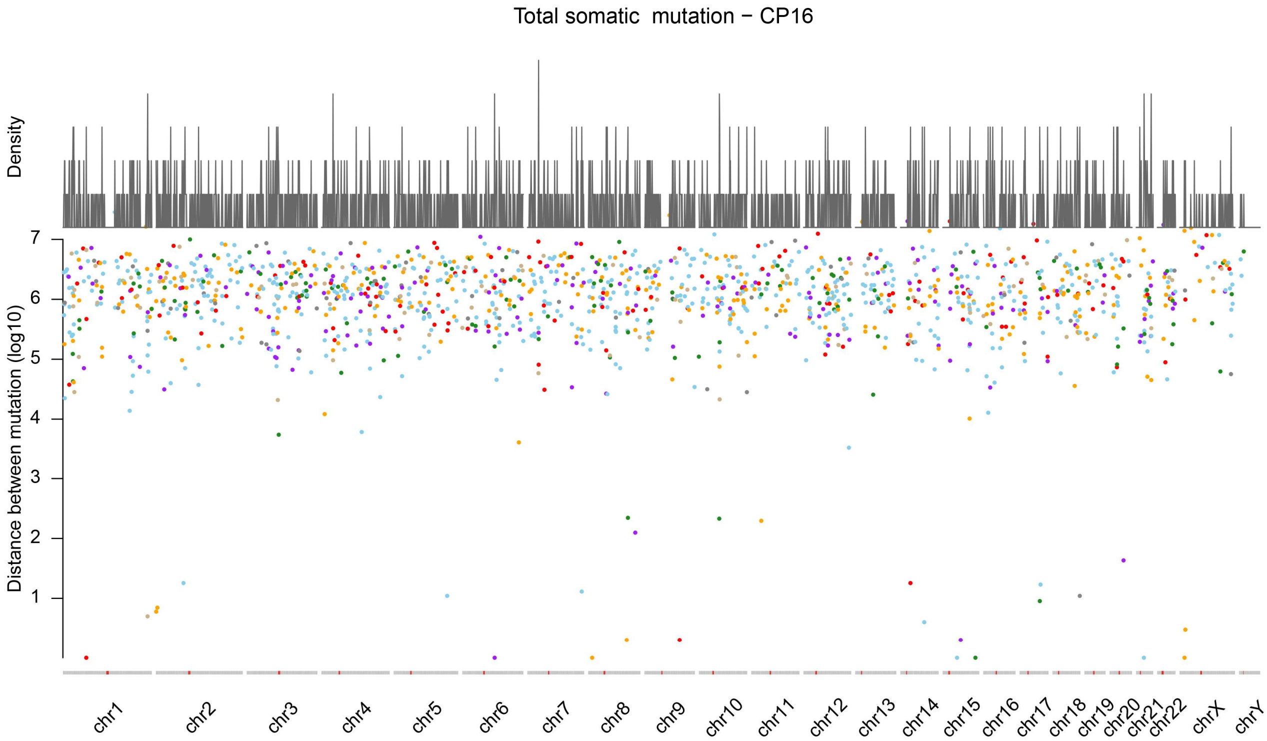
Total somatic mutation − CP16
Density
Distance between mutation (log10)
7
6
5
4
3
2
1
chr1
chr2
chr3
chr4
chr5
chr6
chr7
chr8
chr9
chr10
chr11
chr12
chr13
chr14
chr15
chr16
chr17
chr18
chr19
chr20
chr21
chr22
chrX
chrY

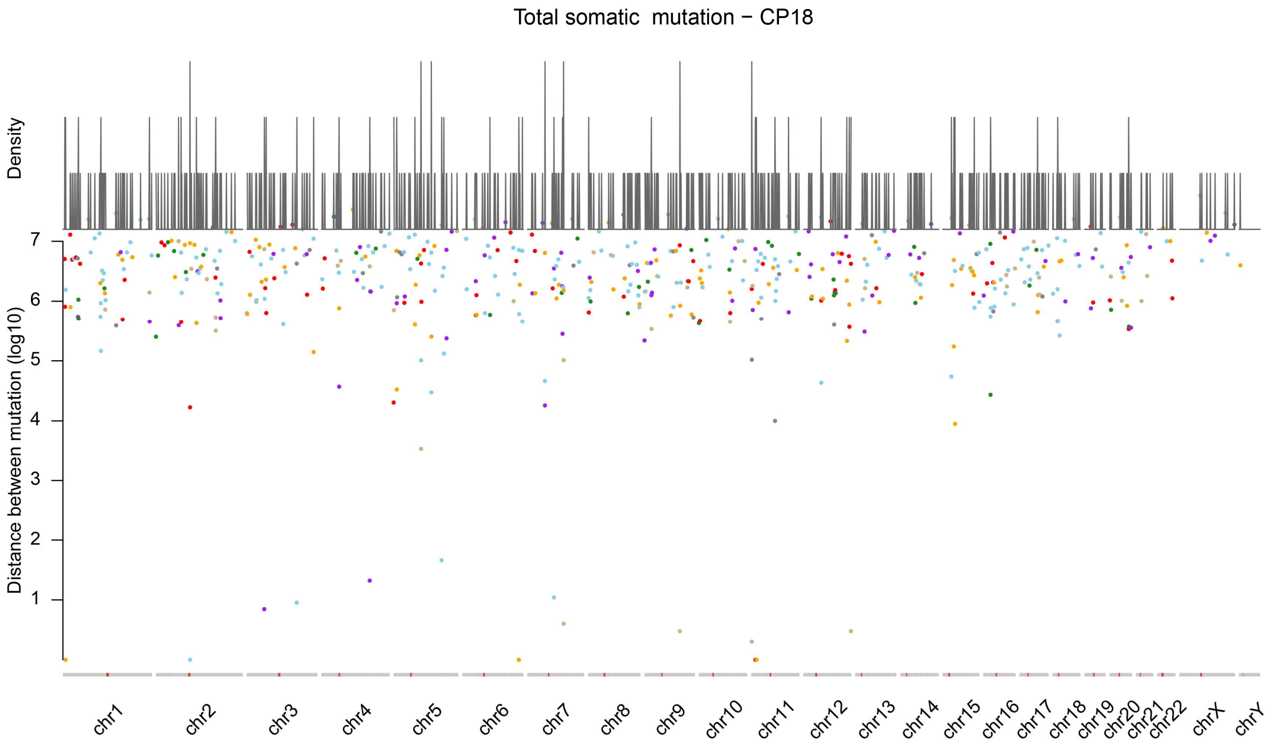
Total somatic mutation − CP18
Density
Distance between mutation (log10)
7
6
5
4
3
2
1
chr1
chr2
chr3
chr4
chr5
chr6
chr7
chr8
chr9
chr10
chr11
chr12
chr13
chr14
chr15
chr16
chr17
chr18
chr19
chr20
chr21
chr22
chrX
chrY

Total somatic mutation − CP19

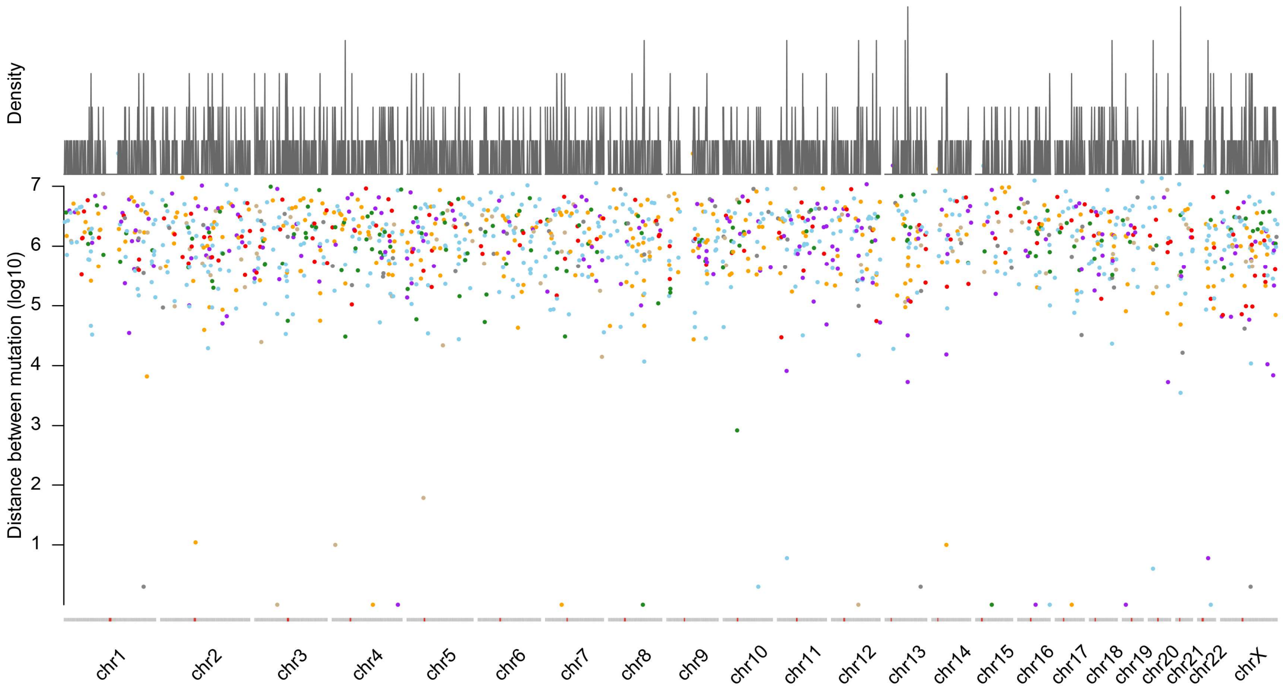

Total somatic mutation − CP20

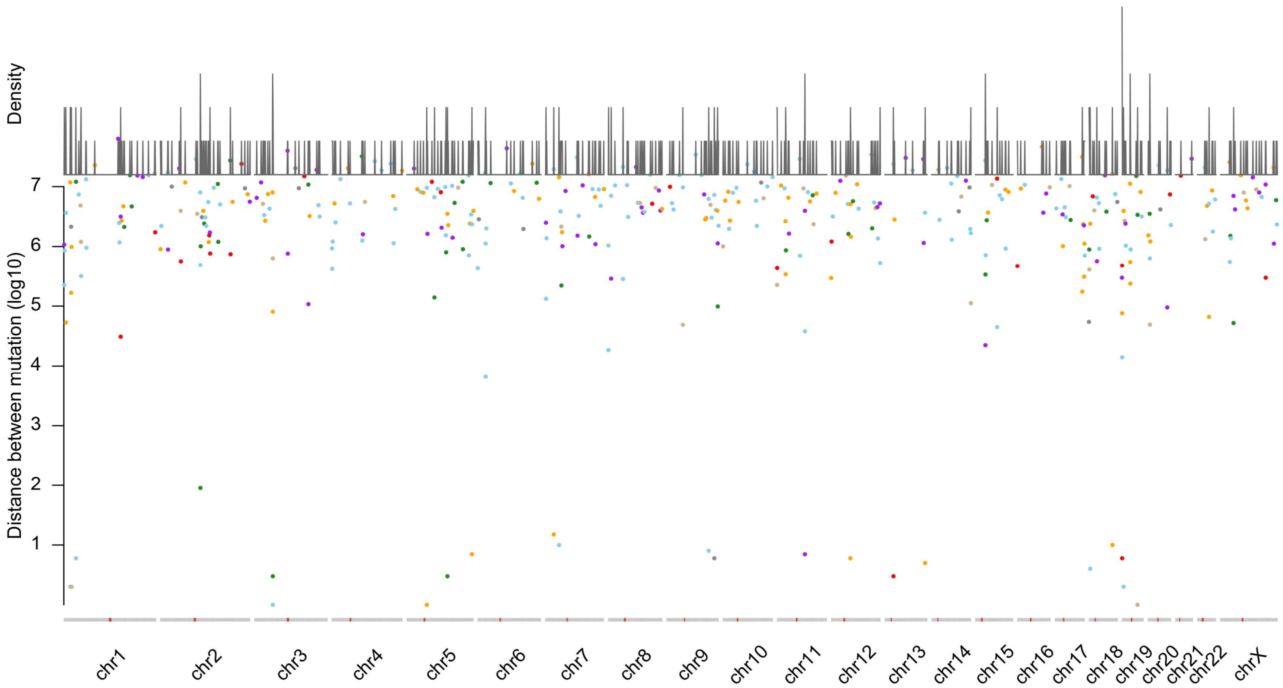

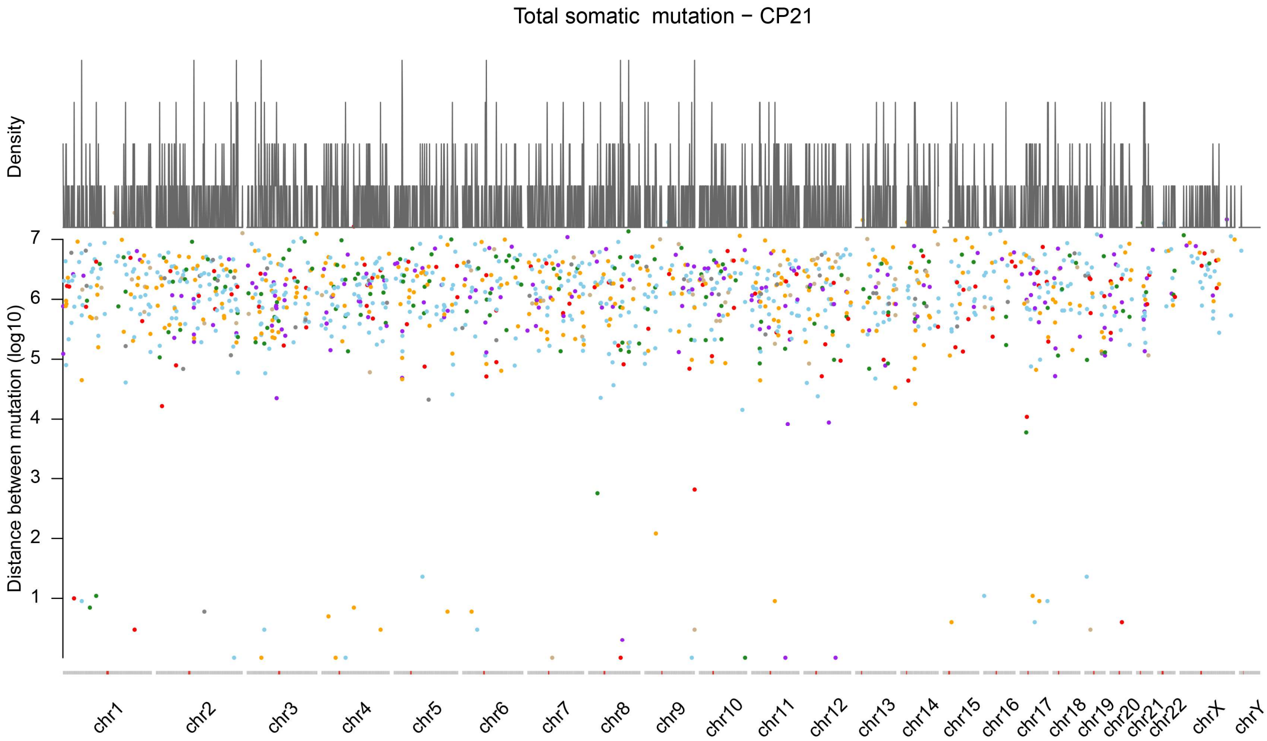

Total somatic mutation − CP21
Density
Distance between mutation (log10)
7
6
5
4
3
2
1
chr1
chr2
chr3
chr4
chr5
chr6
chr7
chr8
chr9
chr10
chr11
chr12
chr13
chr14
chr15
chr16
chr17
chr18
chr19
chr20
chr21
chr22
chrX
chrY

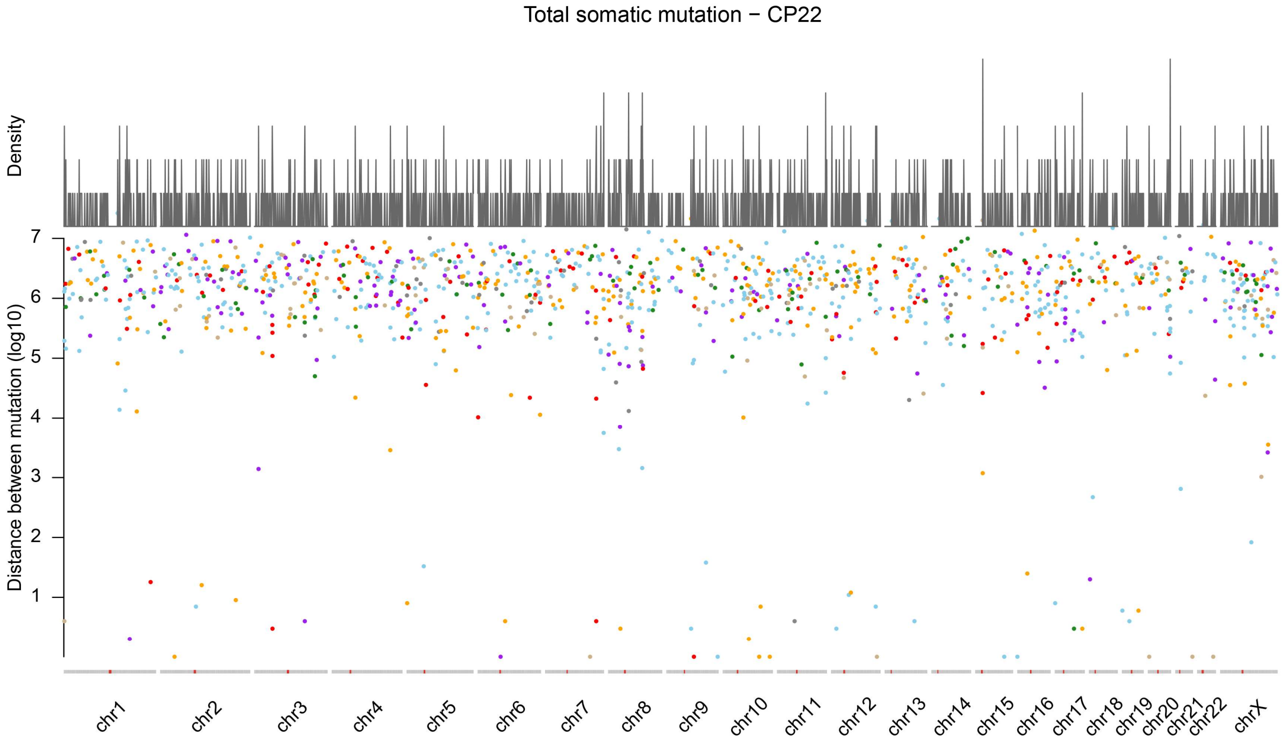

Total somatic mutation − CP22
Density
Distance between mutation (log10)
7
6
5
4
3
2
1
chr1
chr2
chr3
chr4
chr5
chr6
chr7
chr8
chr9
chr10
chr11
chr12
chr13
chr14
chr15
chr16
chr17
chr18
chr19
chr20
chr21
chr22
chrX

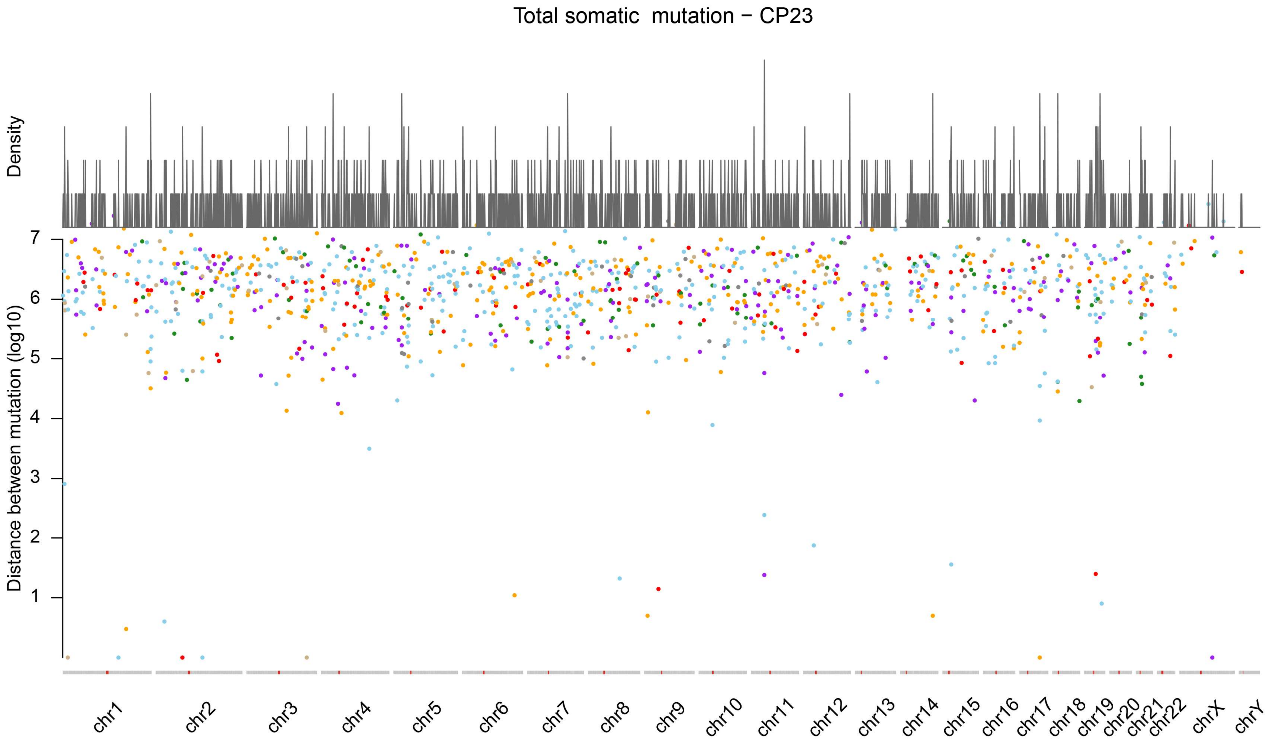
Total somatic mutation − CP23
Density
Distance between mutation (log10)
7
6
5
4
3
2
1
chr1
chr2
chr3
chr4
chr5
chr6
chr7
chr8
chr9
chr10
chr11
chr12
chr13
chr14
chr15
chr16
chr17
chr18
chr19
chr20
chr21
chr22
chrX
chrY

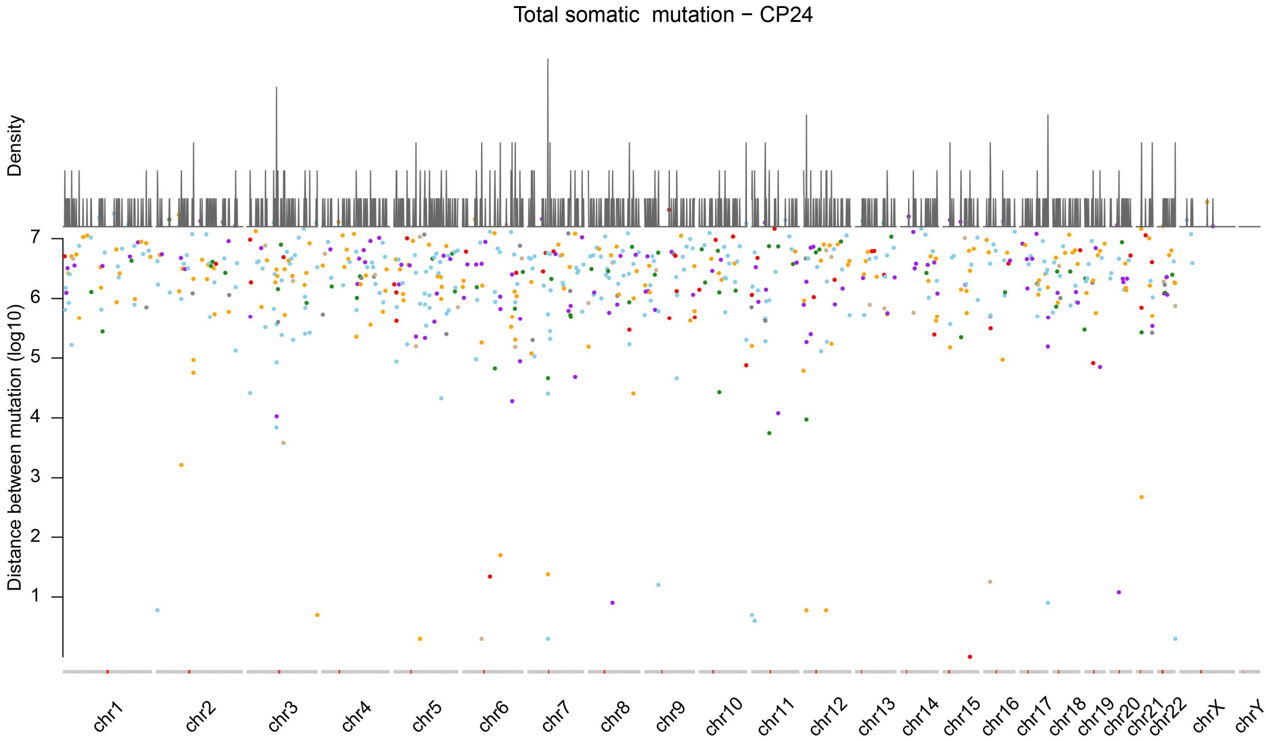
Total somatic mutation − CP24
Density
Distance between mutation (log10)
7
6
5
4
3
2
1
chr1
chr2
chr3
chr4
chr5
chr6
chr7
chr8
chr9
chr10
chr11
chr12
chr13
chr14
chr15
chr16
chr17
chr18
chr19
chr20
chr21
chr22
chrX
chrY

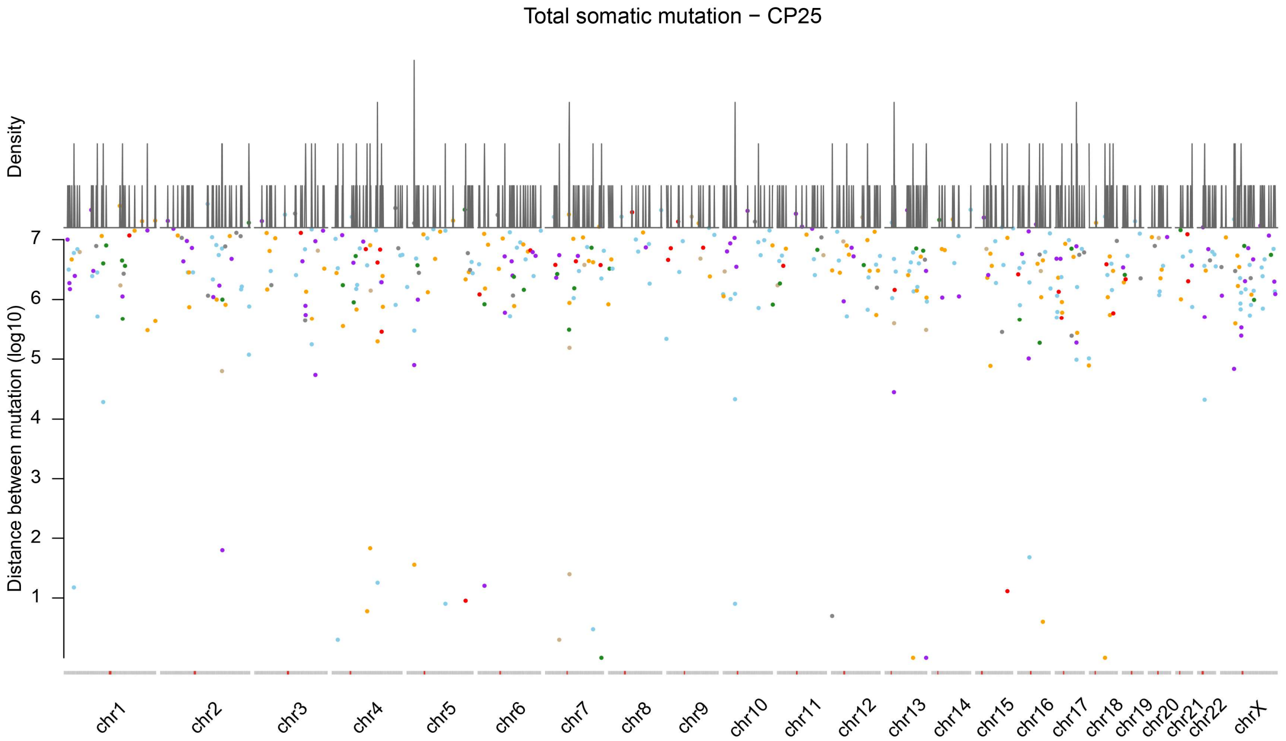

Total somatic mutation − CP25
Density
Distance between mutation (log10)
7
6
5
4
3
2
1
chr1
chr2
chr3
chr4
chr5
chr6
chr7
chr8
chr9
chr10
chr11
chr12
chr13
chr14
chr15
chr16
chr17
chr18
chr19
chr20
chr21
chr22
chrX

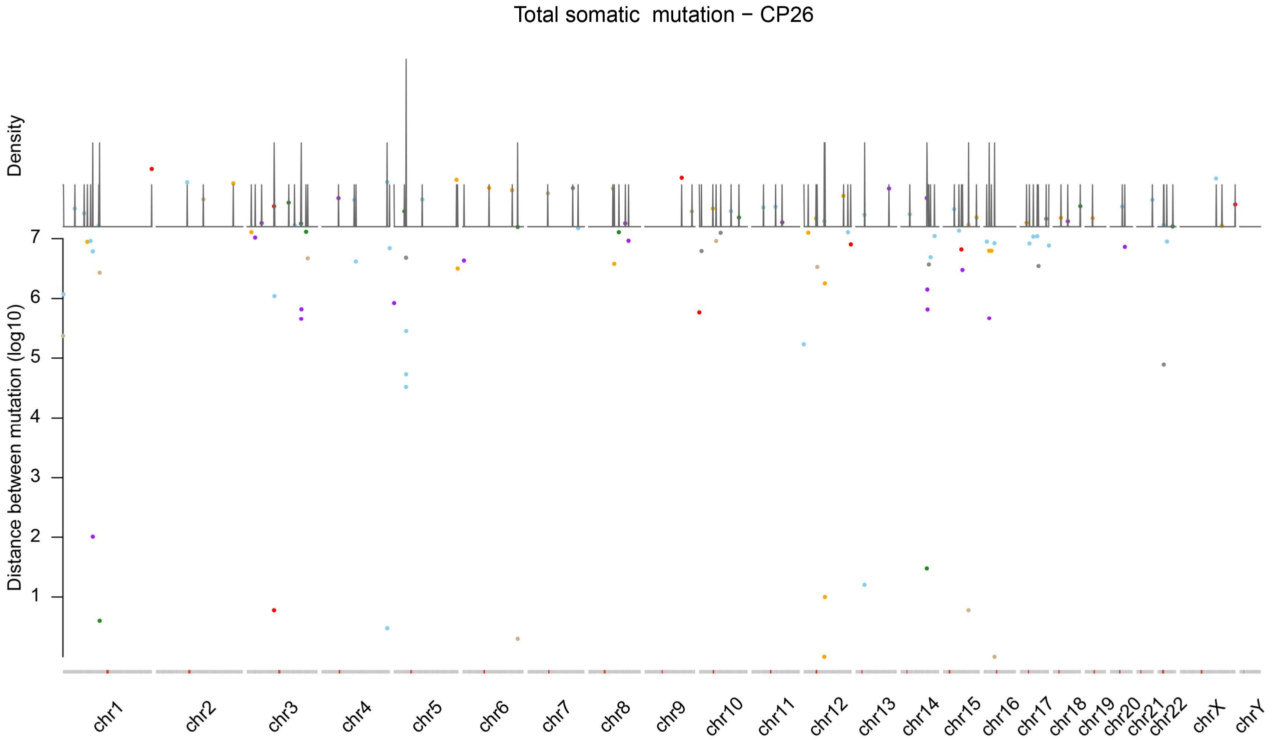

Total somatic mutation − CP26
Density
Distance between mutation (log10)
7
6
5
4
3
2
1
chr1
chr2
chr3
chr4
chr5
chr6
chr7
chr8
chr9
chr10
chr11
chr12
chr13
chr14
chr15
chr16
chr17
chr18
chr19
chr20
chr21
chr22
chrX
chrY

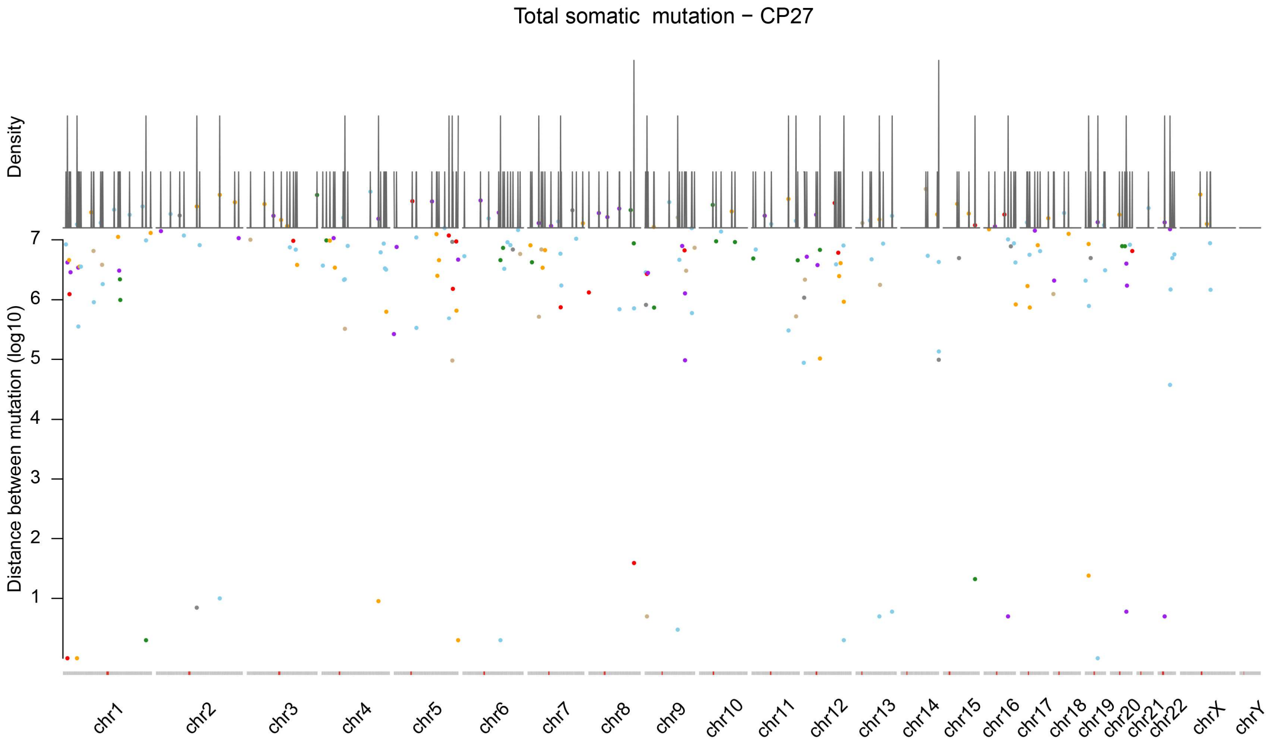
Total somatic mutation − CP27
Density
Distance between mutation (log10)
7
6
5
4
3
2
1
chr1
chr2
chr3
chr4
chr5
chr6
chr7
chr8
chr9
chr10
chr11
chr12
chr13
chr14
chr15
chr16
chr17
chr18
chr19
chr20
chr21
chr22
chrX
chrY

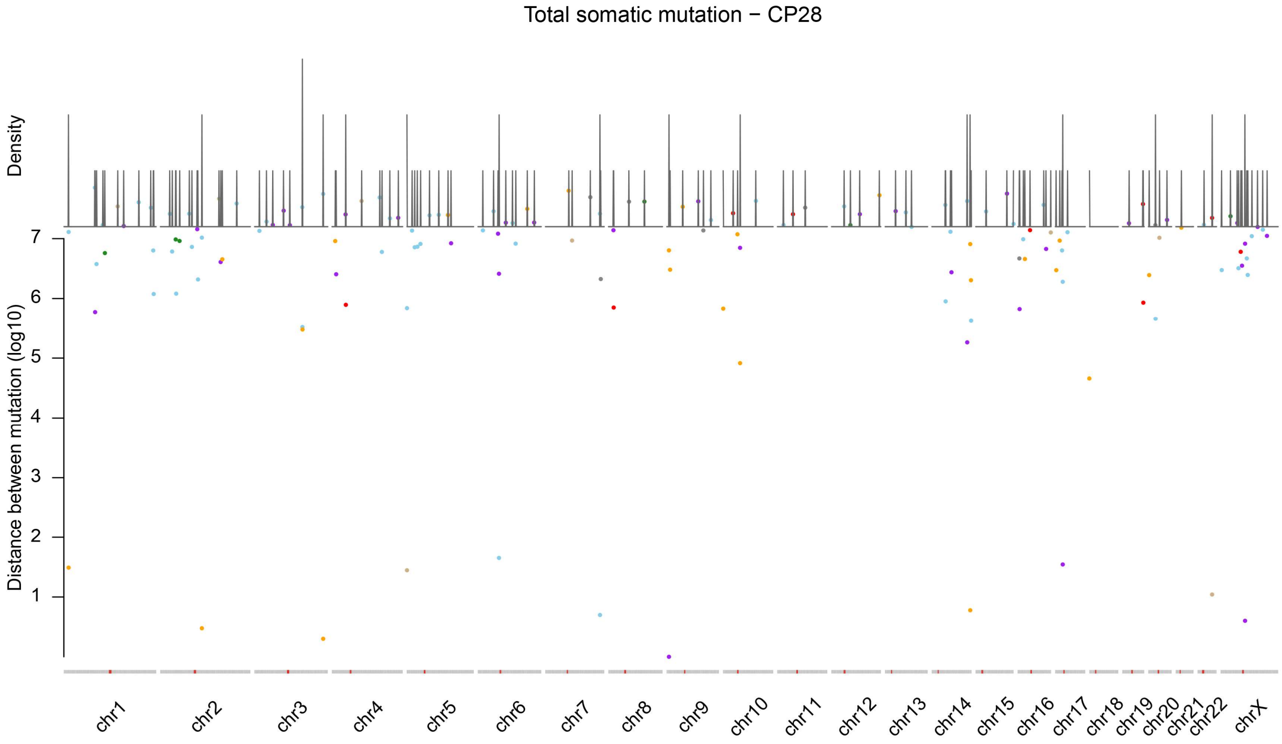
Total somatic mutation − CP28
Density
Distance between mutation (log10)
7
6
5
4
3
2
1
chr1
chr2
chr3
chr4
chr5
chr6
chr7
chr8
chr9
chr10
chr11
chr12
chr13
chr14
chr15
chr16
chr17
chr18
chr19
chr20
chr21
chr22
chrX

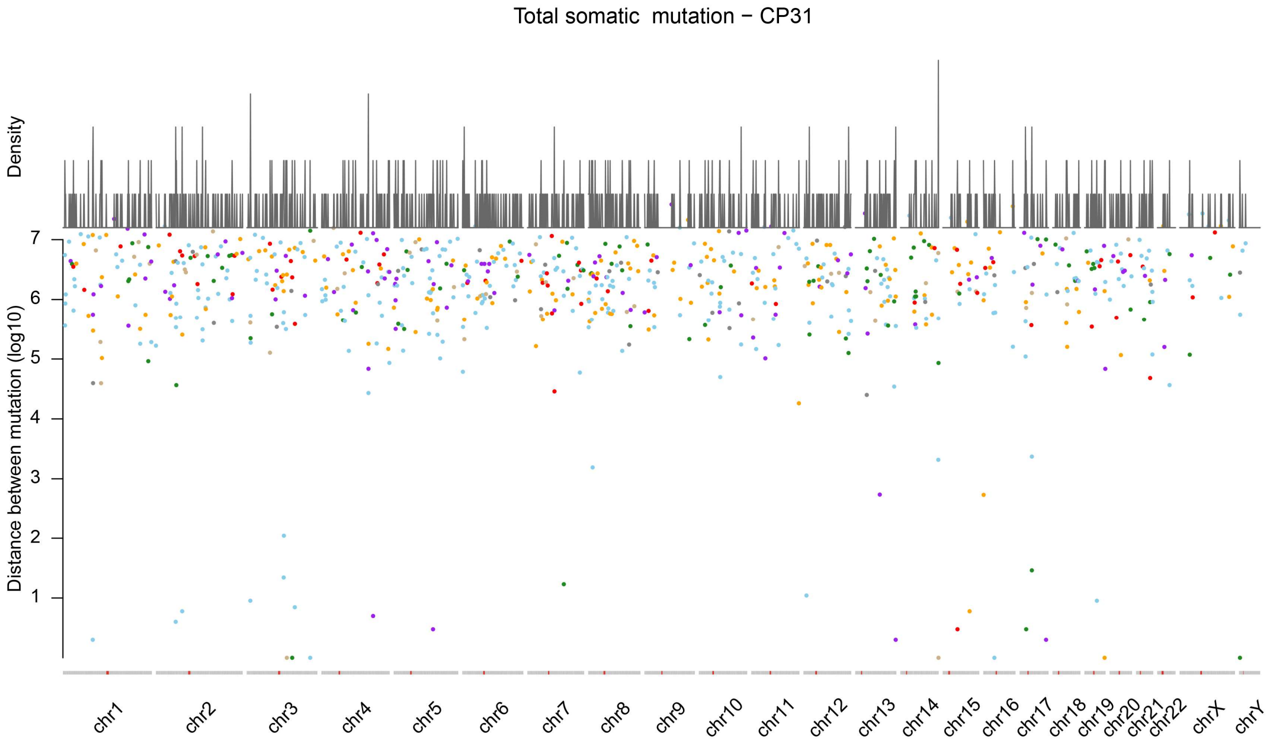
Total somatic mutation − CP31
Density
Distance between mutation (log10)
7
6
5
4
3
2
1
chr1
chr2
chr3
chr4
chr5
chr6
chr7
chr8
chr9
chr10
chr11
chr12
chr13
chr14
chr15
chr16
chr17
chr18
chr19
chr20
chr21
chr22
chrX
chrY

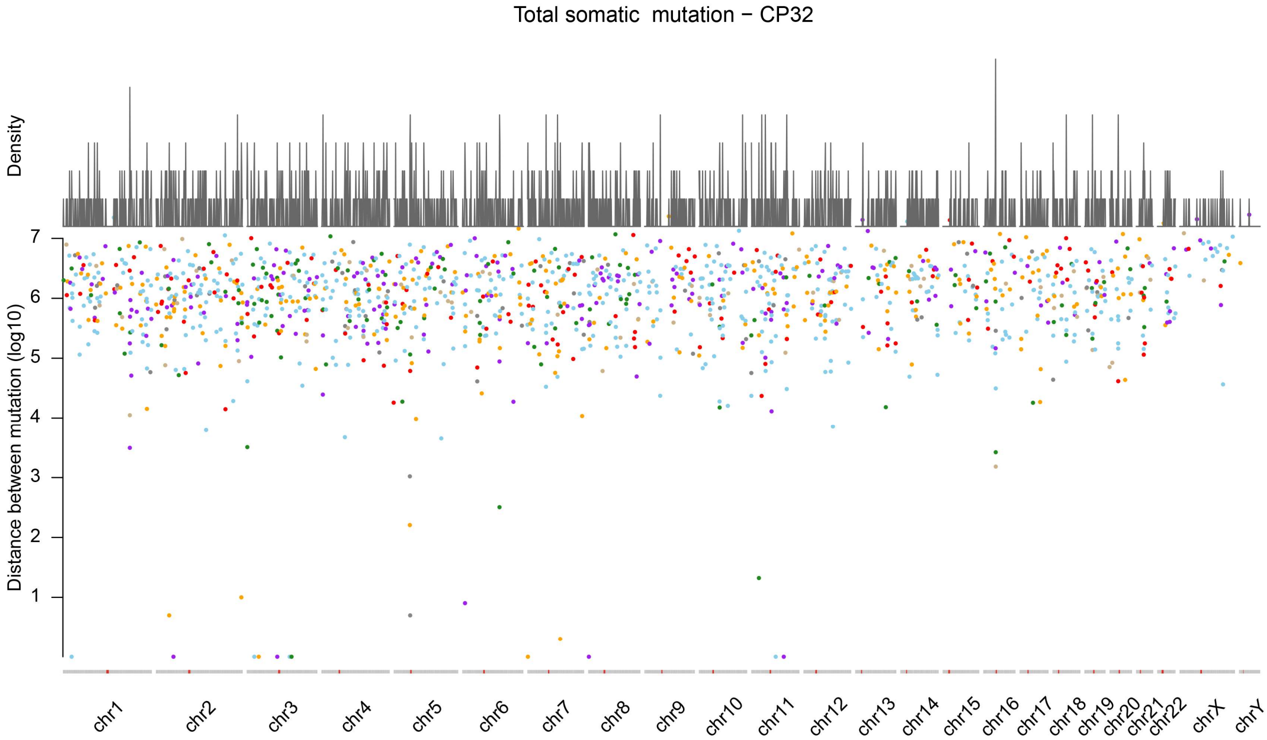
Total somatic mutation − CP32
Density
Distance between mutation (log10)
7
6
5
4
3
2
1
chr1
chr2
chr3
chr4
chr5
chr6
chr7
chr8
chr9
chr10
chr11
chr12
chr13
chr14
chr15
chr16
chr17
chr18
chr19
chr20
chr21
chr22
chrX
chrY

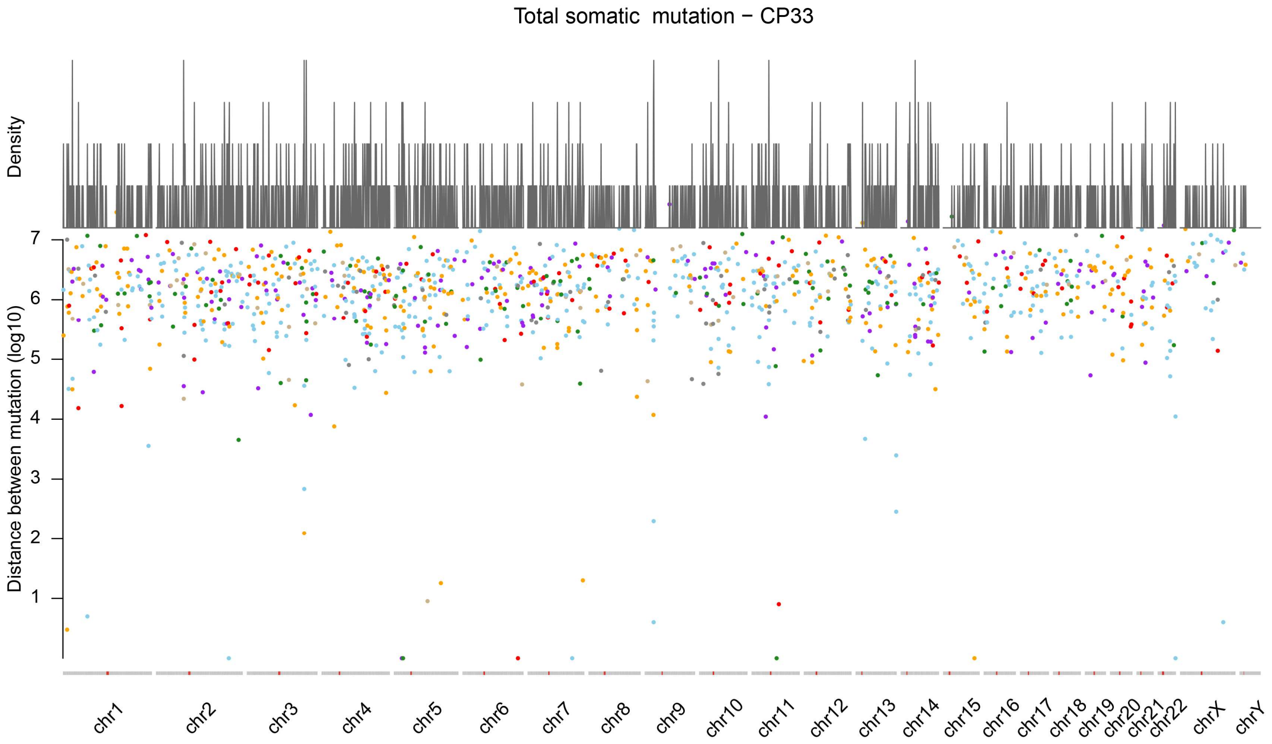
Total somatic mutation − CP33
Density
Distance between mutation (log10)
1
2
3
4
5
6
7
chr1
chr2
chr3
chr4
chr5
chr6
chr7
chr8
chr9
chr10
chr11
chr12
chr13
chr14
chr15
chr16
chr17
chr18
chr19
chr20
chr21
chr22
chrX
chrY

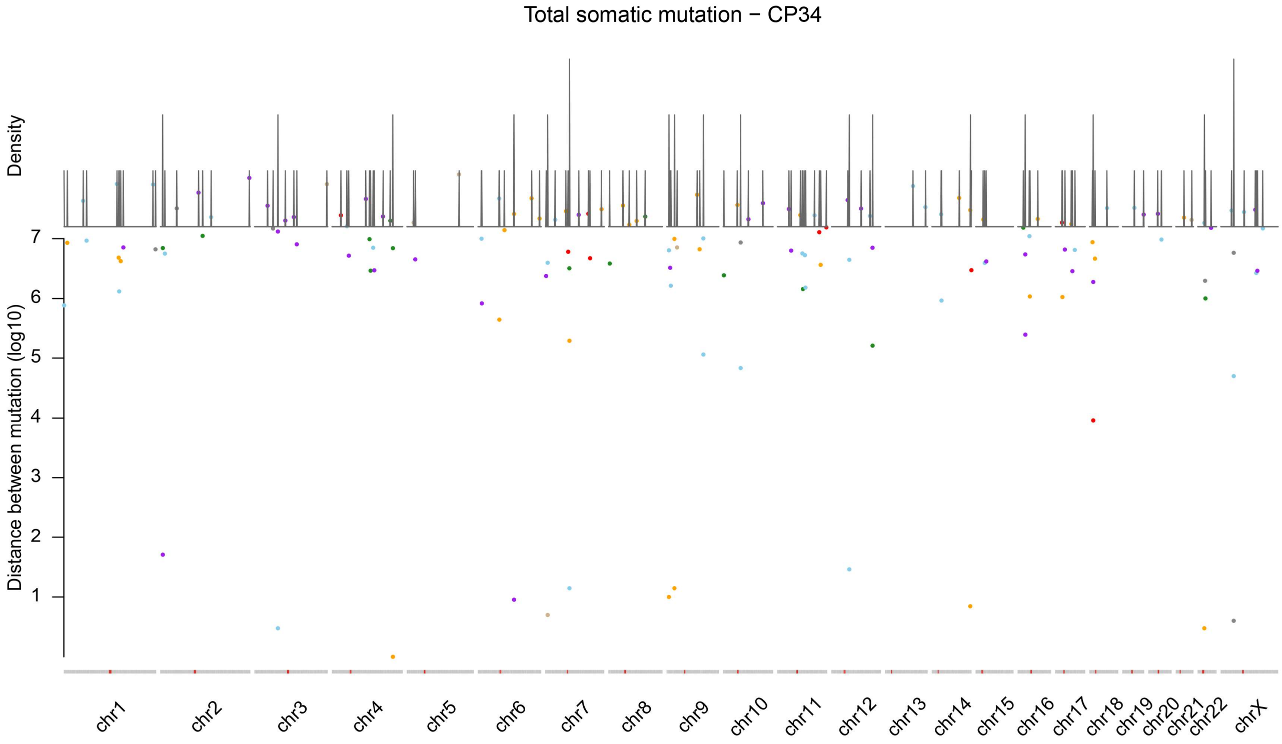
Total somatic mutation − CP34
Density
Distance between mutation (log10)
1
2
3
4
5
6
7
chr1
chr2
chr3
chr4
chr5
chr6
chr7
chr8
chr9
chr10
chr11
chr12
chr13
chr14
chr15
chr16
chr17
chr18
chr19
chr20
chr21
chr22
chrX

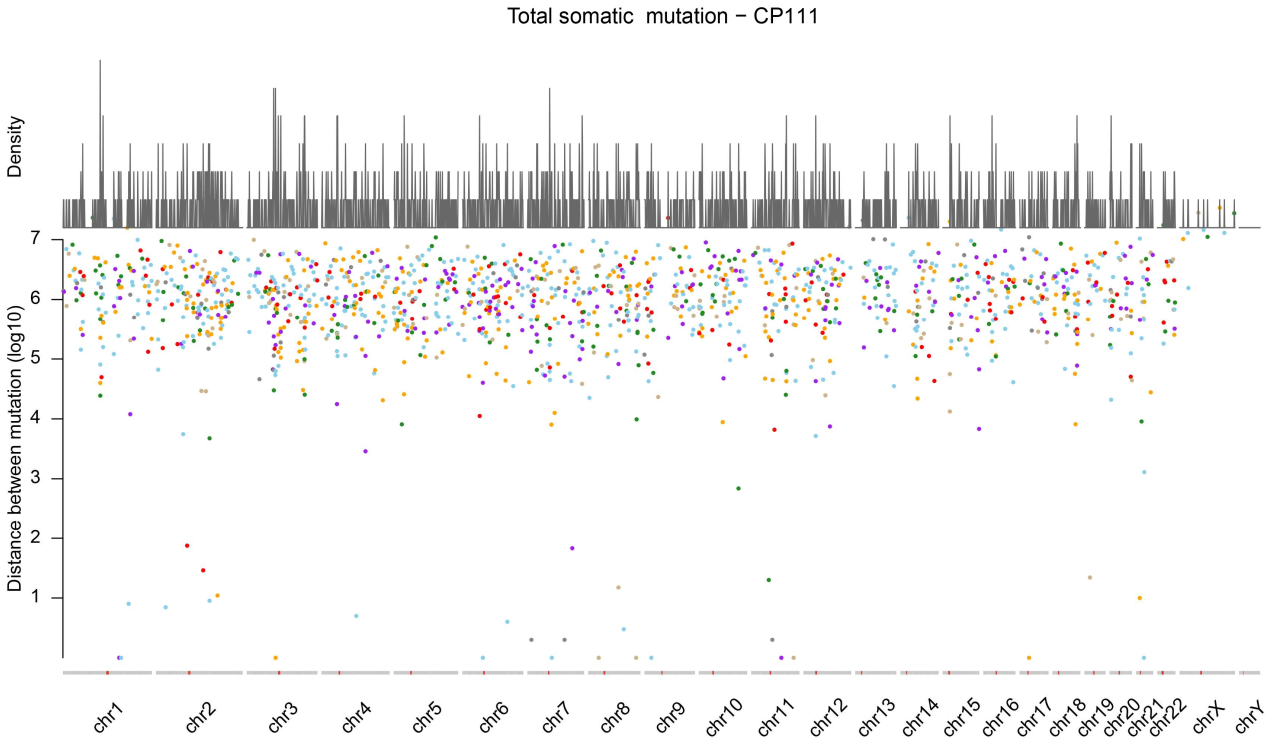
Total somatic mutation − CP111
Density
Distance between mutation (log10)
chr1 chr2 chr3 chr4 chr5 chr6 chr7 chr8 chr9 chr10 chr11 chr12 chr13 chr14 chr15 chr16 chr17 chr18 chr19 chr20 chr21 chr22 chrX chrY

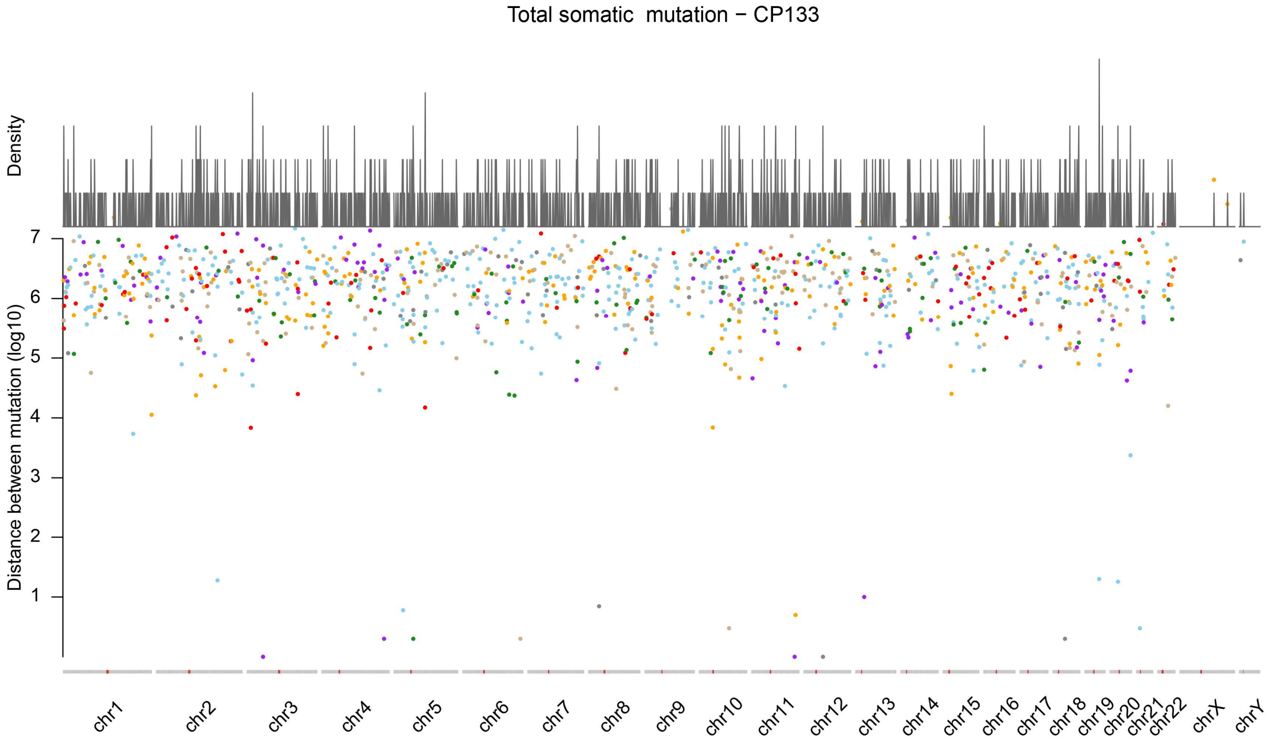
Total somatic mutation − CP133
Density
Distance between mutation (log10)
chr1 chr2 chr3 chr4 chr5 chr6 chr7 chr8 chr9 chr10 chr11 chr12 chr13 chr14 chr15 chr16 chr17 chr18 chr19 chr20 chr21 chr22 chrX chrY

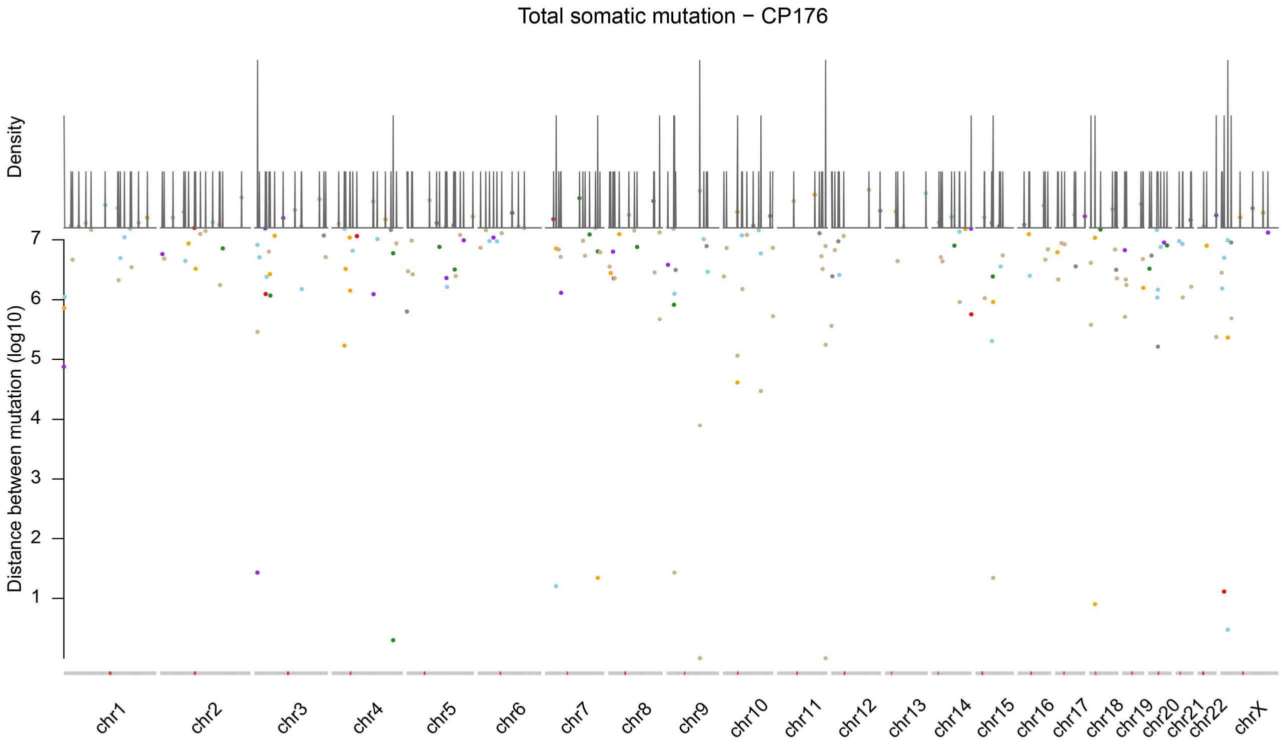
Total somatic mutation − CP176
Density
Distance between mutation (log10)
7
6
5
4
3
2
1
chr1
chr2
chr3
chr4
chr5
chr6
chr7
chr8
chr9
chr10
chr11
chr12
chr13
chr14
chr15
chr16
chr17
chr18
chr19
chr20
chr21
chr22
chrX

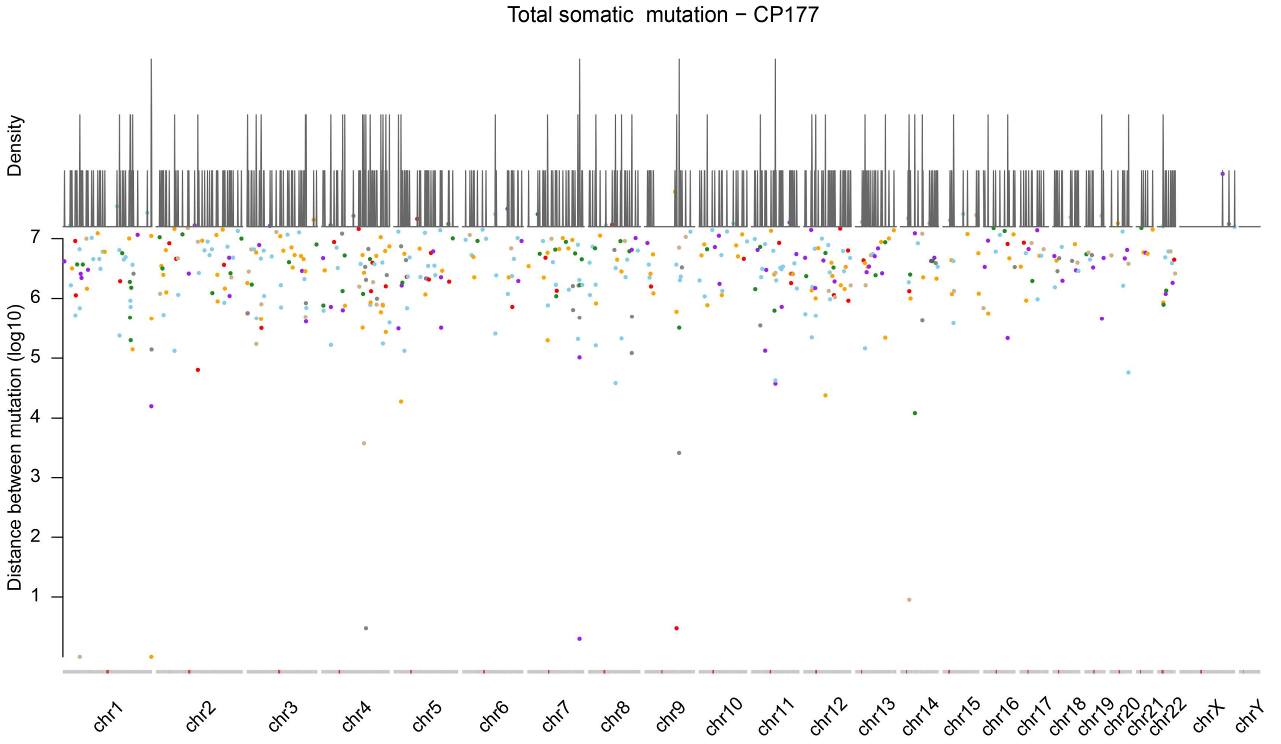
Total somatic mutation − CP177
Density
Distance between mutation (log10)
7
6
5
4
3
2
1
chr1
chr2
chr3
chr4
chr5
chr6
chr7
chr8
chr9
chr10
chr11
chr12
chr13
chr14
chr15
chr16
chr17
chr18
chr19
chr20
chr21
chr22
chrX
chrY

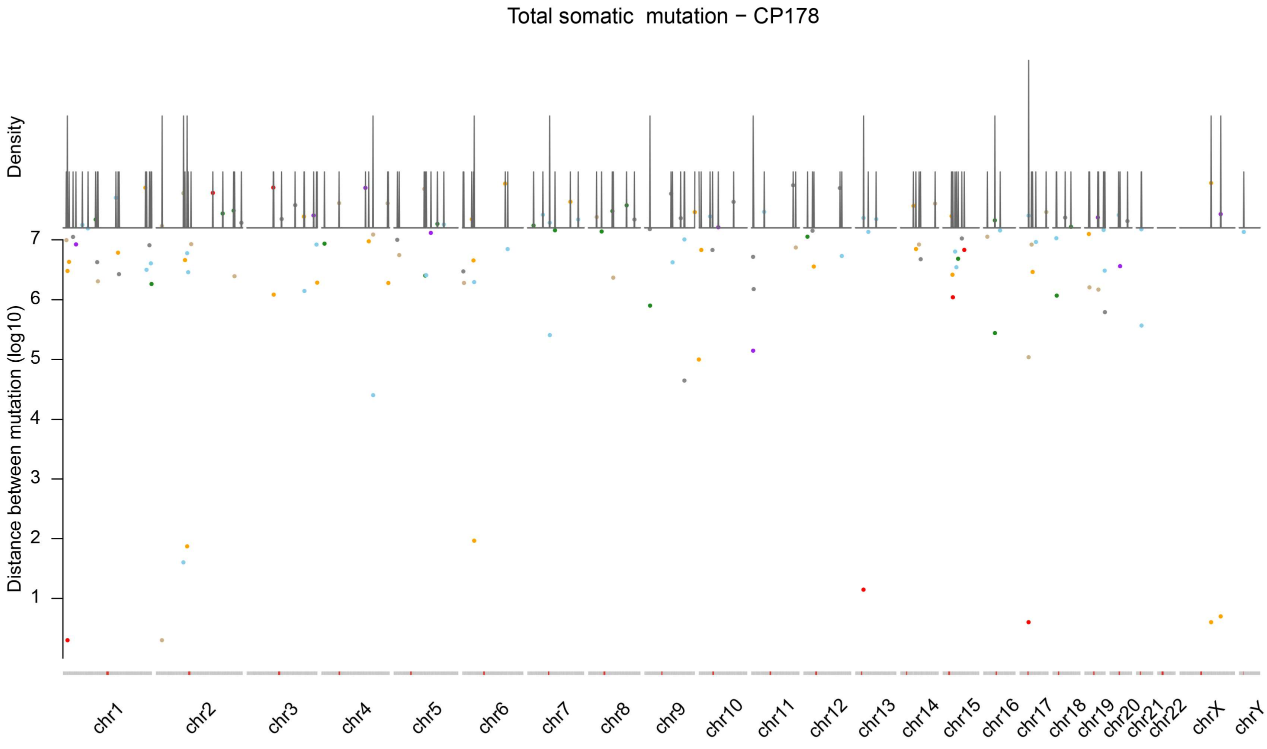

Fig S4. Kataegis analysis of somatic mutations (SNVs and InDels) in each patient.

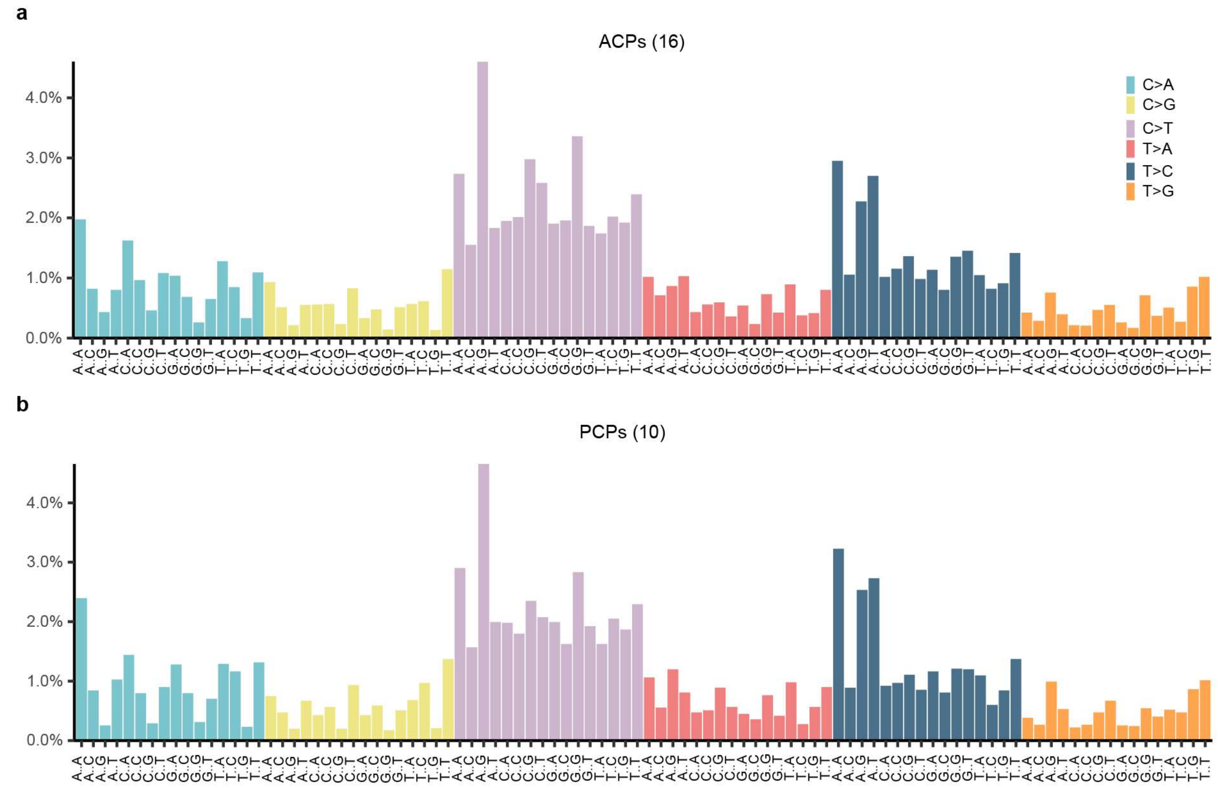

Fig S5. The proportion of 96 different extending possible base-pair substitutions of all SNVs identified in 16 ACPs and 10 PCPs.

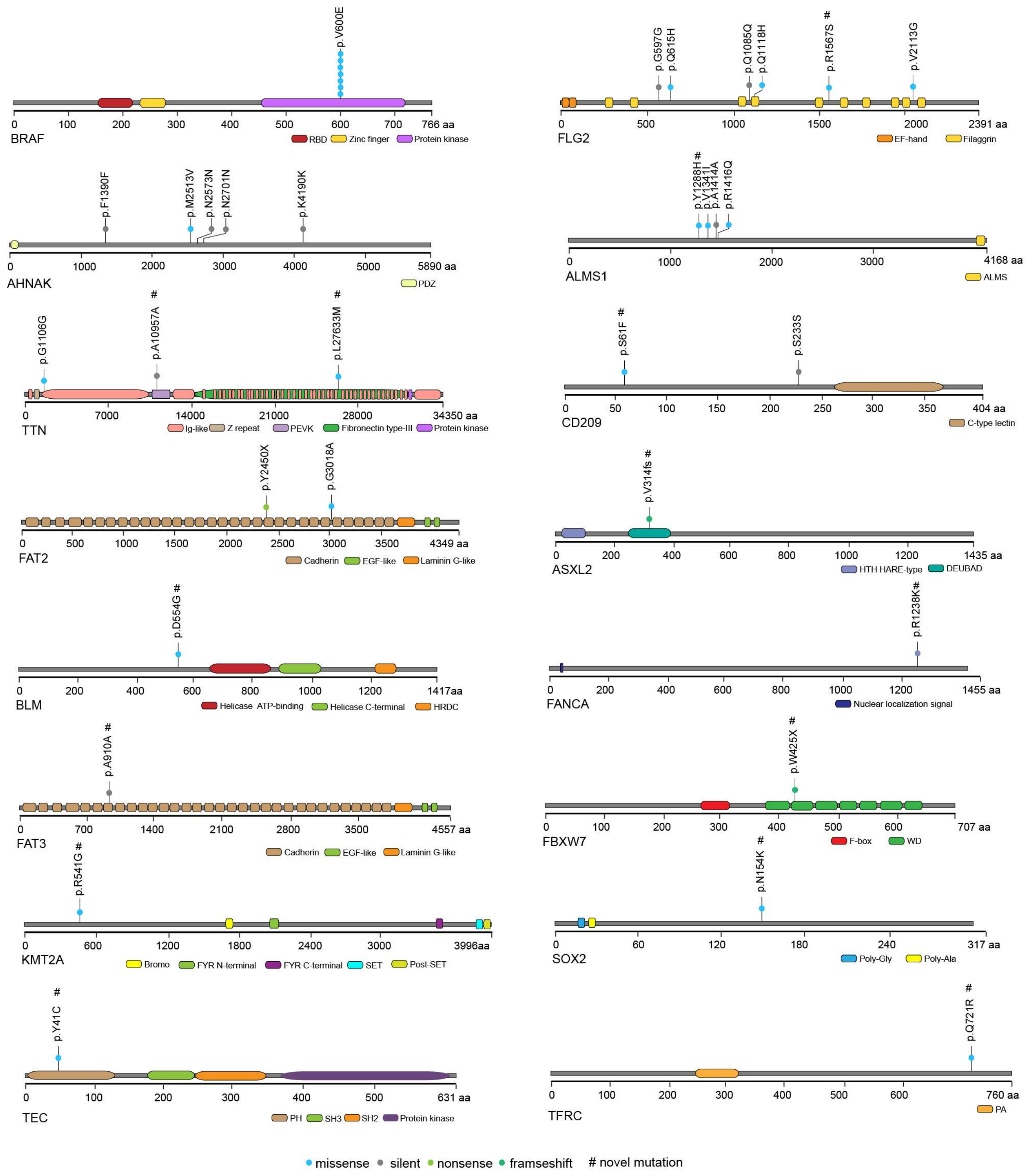

Fig S6. Schematics for genes listed in Fig. 3a indicating the location of the identified mutations.

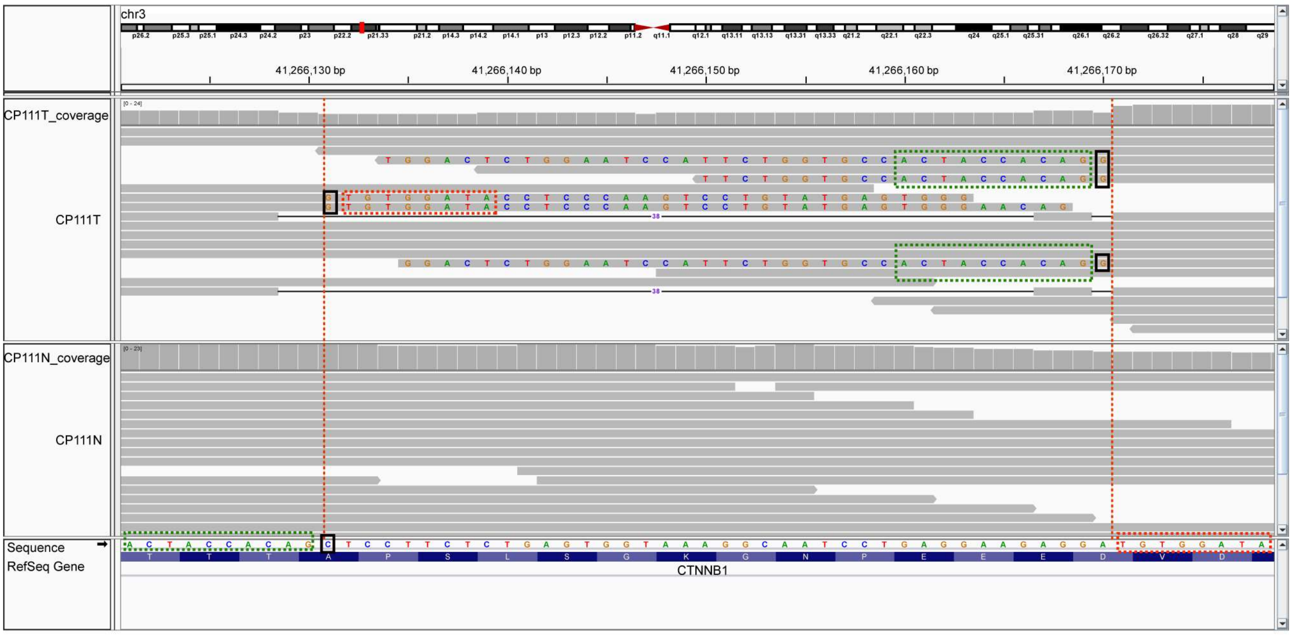

Fig S7. IGV graphic report for the alignment of deletion in CTNNB1 CP111 tumor and normal blood sample.

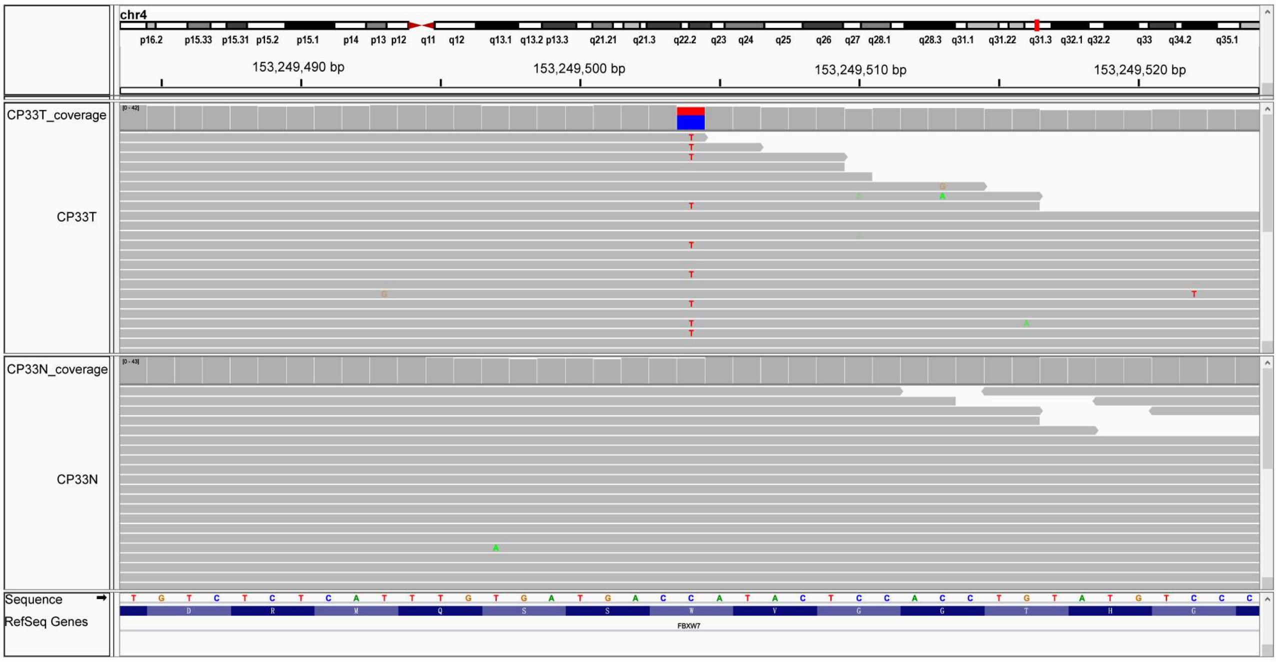

Fig S8. IGV graphic report for the alignment of nonsense mutation of W425X in FBXW7 of CP22 tumor and normal blood sample.

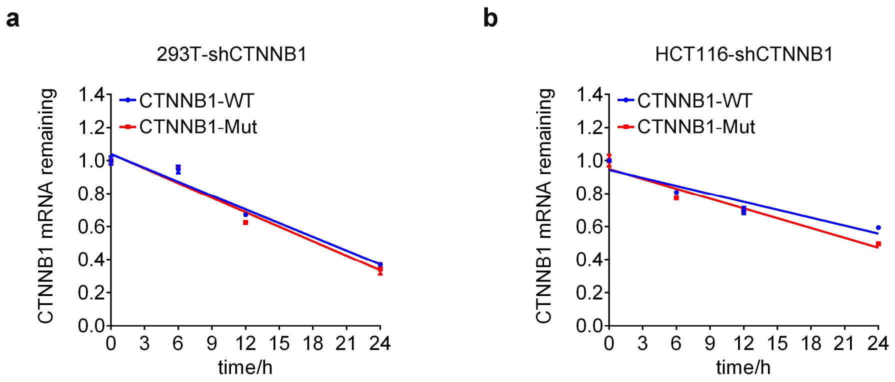

Fig S9. CTNNB1 mRNA stability in CTNNB1-WT/Mut 293T/HCT116 cells. a-b CTNNB1 mRNA stability in CTNNB1-WT/Mut 293T/HCT116 treated with 5 μg/mL actinomycin D at different time points. Actin mRNA was used as a negative control.
